# Behavioural economics in fisheries: A systematic review protocol

**DOI:** 10.1371/journal.pone.0255333

**Published:** 2021-08-26

**Authors:** Alina M. Wieczorek, Amanda Schadeberg, Julie Krogh Hallin, Ingrid van Putten, Sarah B. M. Kraak, Andries Richter, Patricia M. Clay, Leyre Goti Aralucea, Debbi Pedreschi, Katell G. Hamon, Dorothy J. Dankel, Mary Mackay

**Affiliations:** 1 Marine Institute, Oranmore, Co. Galway, Ireland; 2 Wageningen University & Research, Environmental Economics and Natural Resources Group, Wageningen, The Netherlands; 3 Wageningen University & Research, Environmental Policy Group, Wageningen, The Netherlands; 4 ICES Secretariat, Copenhagen V, Denmark; 5 CSIRO Oceans and Atmosphere, Hobart, Australia; 6 Centre for Marine Socio-Ecology, University of Tasmania, Hobart, Australia; 7 Thünen Institute of Baltic Sea Fisheries, Rostock, Germany; 8 CEES, Department of Biosciences, University of Oslo, Oslo, Norway; 9 NOAA Fisheries, Silver Spring, MD, United States of America; 10 Thünen Institute of Sea Fisheries, Bremerhaven, Germany; 11 Wageningen Economic Research, Wageningen, The Netherlands; 12 Department of Biological Sciences, University of Bergen, Bergen, Norway; 13 Nordic Marine Think Tank, nmtt.org, Norway; University of Florida, UNITED STATES

## Abstract

**Background:**

The field of behavioural economics holds several opportunities for integrated fisheries management and conservation and can help researchers and managers alike understand fisher behaviour and decision-making. As the study of the cognitive biases that influence decision-making processes, behavioural economics differentiates itself from the classical field of economics in that it does not assume strictly rational behaviour of its agents, but rather looks for all mechanisms that influence behaviour. This field offers potential applications for fisheries management, for example in relation to behavioural change, but such applications require evidence of these mechanisms applied in a fisheries context. Thus, we have developed a systematic literature review protocol focusing on the primary question: “Which behavioural economics mechanisms influence fisher behaviour?” The aim is to provide a comprehensive overview of these different mechanisms and how they have been applied in the study of fisher behaviour.

**Methods and expected outputs:**

The review protocol was developed in close collaboration with the International Council for the Exploration of the Sea (ICES) Working Group on Maritime Systems (WGMARS). WGMARS members were therefore considered the key stakeholders for this study, and were consulted to develop a suitable systematic review question and methodology. Three academic databases will be searched using a customized Boolean keyword search string. Research articles deemed eligible for inclusion in the systematic review are those that studied the influence of behavioural-economics mechanisms on the behaviour of marine fishers in any location, and at any scale. Insights from this literature will be collated in order to provide an overview of the relevant behavioural-economics mechanisms and actions, how effective these mechanisms are and at what scale, geographic region and in which fisheries sector they have been applied. Any fisheries management implications identified by the studies under review will also be outlined. Finally, it will be recorded whether or not ethical considerations were made in the reviewed literature, so that in the discussion it will be possible to reflect on the ethics of conducting behavioural-economics research and policy actions in a fisheries context.

## Introduction

### Rationale

Human behaviour is a key source of uncertainty in fisheries management [1] and management institutions are therefore increasingly interested in understanding human behaviour. The International Council for the Exploration of the Seas (ICES) is a science and advice-giving inter-governmental organization that informs evidence-based fisheries management. The ICES expert Working Group on Maritime Systems (WGMARS) produces research about human behaviour in fisheries that is relevant for governing bodies. WGMARS has set their focus for the 2020–2022 period on methodological, operational, contextual, and management aspects that enable successful ecosystem-based marine management and governance. As part of their terms of reference for this period, WGMARS aims to analyse how the use of behavioural economics can support integrated ecosystem assessment and ecosystem-based management [2]. Behavioural economics studies the cognitive biases and other drivers/mechanisms in decision-making processes. In contrast to traditional economics, which typically neglects the social complexity and context-dependency of human decision-making, behavioural economics makes use of a richer toolbox to explain human behaviour. In fisheries, a top-down management approach under the presumption of rational profit-maximising decision making by fishers is widely employed. While this is effective to an extent, such an approach omits other important mechanisms that are part of fisher behaviour. This may lead to unintended outcomes and raises uncertainties around the success of fisheries-management approaches [1, 3]. Some initial studies have explored the potential of using insights from behavioural economics in a fisheries context (e.g., [4, 5]). The proposed systematic review aims to consolidate knowledge from diverse literature on the use of behavioural-economics mechanisms in a fisheries context and provide an overview of relevant findings. Such an overview will be crucial for guiding the actions and recommendations within the ICES WGMARS and for others interested in understanding human behaviour in a fisheries management context.

### Stakeholder engagement

The need for evidence on the use of behavioural economics in fisheries was identified by the behavioural-economics task group of WGMARS (BE task group hereafter). This group commissioned the review study by advertising the work to early-career researchers through their various networks. Three suitable researchers (lead authors hereafter) with heterogeneous backgrounds were identified:

- Alina Madita Wieczorek (AMW) is a post-doctoral researcher focusing on mesopelagic fisheries acoustics and ecology, with a background in marine microplastic research and an interest in science communication.
- Amanda Schadeberg (AS) is a PhD candidate in mesopelagic fisheries governance, with a background in studying fisher behaviour using social-science methods.
- Julie Krogh Hallin (JKH) has a MSc in Nature Management and is working for ICES Secretariat in the Science Department with a focus on the Integrated Ecosystem Assessment Steering Group’s expert groups, science communication, symposia planning and project collaboration.

The three lead authors dedicate their time out of interest in the topic, to gain experience in conducting such a study and to closely interact with and learn from the experts in the WGMARS.

**Fig 1:**
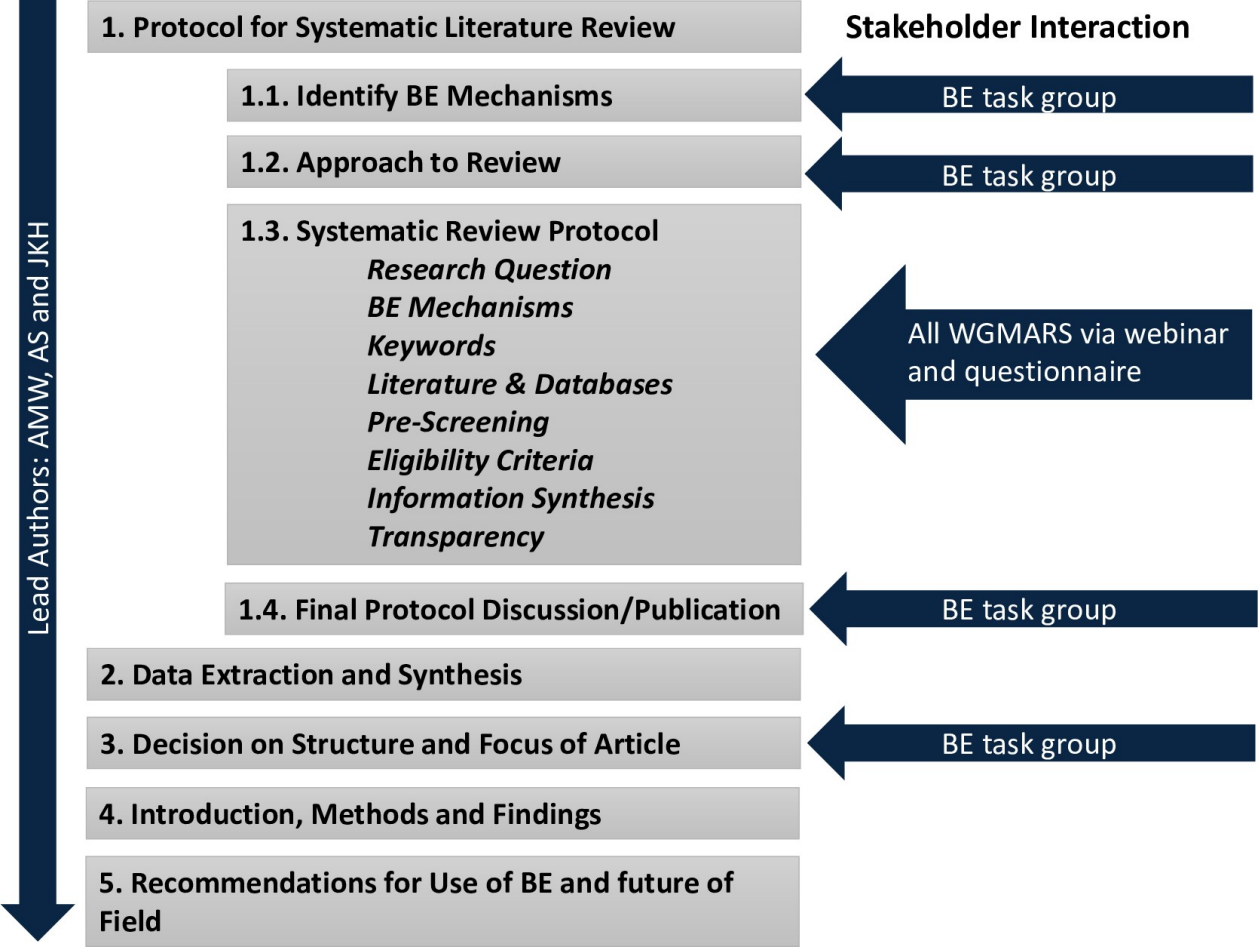
Flow chart depicting the design of the systematic review, indicating the moment where input from the BE task group and WGMARS members was solicited.

First, the lead authors established the scope of the review (Table 1, Fig 1). For this study, stakeholders are defined as anyone who would be interested to understand, and/or potentially apply or recommend, the application of, behavioural-economics insights into fisheries management. Thus, as a key link between scientific research and policymakers, the key stakeholders for the scoping and design development of the systematic review are members of WGMARS (S2 Table). The group comprises a mix of natural scientists, economists, and other social scientists. The BE task group was consulted closely while WGMARS members were consulted through a webinar and with a follow-up feedback questionnaire on the scope and design of the systematic review (Table 1, Fig 1, S1 and S2 Files).

**Table 1:** Timeline of stakeholder consultation for the development of this protocol.

Meeting	Timeframe	Participants	What were stakeholders consulted on?
**Initial Scoping Meeting**	15/10/2020	Lead authors	Definition of behavioural economics
		BE task group	Niche of the study?
			Who is the target group?
			What is the desired result?
			Considerations on scale, effectiveness and ethics
			Next steps
			Who are the stakeholders?
**E-mail feedback**	04/11/2020–13/11/2020	Lead authors	List of behavioural-economic mechanisms
		BE task group	Stakeholder involvement
			Primary research question
			Secondary research questions
**Webinar**	25/11/2020	Lead authors	Primary research question
		All WGMARS members	Secondary research questions
			Behavioural-economic mechanisms
			Search keywords
			Primary databases
			Subject-specific databases
			Specialised literature sources
			Stakeholder literature suggestions
			Article-screening process
			Eligibility criteria
			Information synthesis
			Protocol registration and publication
**Questionnaire due**	4/12/2020	All WGMARS members	Individual written responses to all the above questions
**Final Protocol Discussion**	15/12/2020	Lead authors	Protocol presentation
		BE task group	Final feedback on scope and design
			Discussion on protocol registration and publication

Of the 55 WGMARS stakeholders who were invited to give feedback through the questionnaire (S2 Table) 11 responded, most of which (n = 9) belonged to the BE task group. As an interdisciplinary group of stakeholders, five classified themselves as economists, four as other social scientists and three as natural scientists, with some selecting more than one scientific specialty.

When asked about the primary and secondary research question (Q7 and Q8 S2 File), stakeholders were divided about whether the research question should focus on fisheries-management strategies specifically or whether the focus should be on providing a broader overview of behavioural-economics mechanisms in fisheries. Nearly all stakeholders recommended to narrow down the focus in one way or another (e.g, only marine and not freshwater fishing) and to focus on fisher behavior. This is because of many differences in the regulatory, institutional, environmental and economic contexts between freshwater and marine fisheries. Specific suggestions in regard to the secondary questions were to investigate the instigating party, any unintended effects and to look at the relationships between the fishers and managers in the studies (Q8 and Q28 S2 File).

In this questionnaire the stakeholders were also asked to comment on a first overview table of behavioural-economics mechanisms which was previously compiled by the lead authors and the BE task group (Q9 S2 File). Input from this was used to draw up a more advanced version of the table, which will be used by the authors as a tool for the full-text screening (S3 Table). The behavioural-economics mechanism table and the comments provided on it were also used to draw up keywords, in addition to specific suggestions made by stakeholders (Q 10–19 S2 File). The stakeholders were presented with several options for databases from which to draw the literature, but most did not see themselves as experienced enough with systematic reviews to comment. When asked about the eligibility criteria (Q 27 S2 File), the group had a varied understanding of which studies should be included, with some disagreement about to what degree classical economics papers should be included in the review study. Where possible, the lead authors have systematically incorporated the feedback of the stakeholders, attempting to account for the diverse perspectives and priorities of the stakeholder group as well as the feasibility of this systematic review.

### Research questions

**Primary question**.

- Which behavioural-economics mechanisms have been documented in the literature as influencing fisher behaviour?

**Secondary questions**.

- What are the traits of the studies of these mechanisms?
- In which geographical areas are studies investigating behavioural-economics mechanisms carried out?
- At which geographic scale are studies conducted?
- Which fisheries scale (e.g. artisanal, small-scale, large-scale) is studied?
- Which gear types are employed in the fishery of interest?
- Which fish stocks are targeted in the fishery of interest?
- Do the studies focus on fishers of particularly depleted fish stocks?
- What factor types outlined by Andrews et al. [5] were found to affect fisher behavior?
- Which behavioural-economics mechanisms and types of interventions have been investigated?
- Who were the instigating parties of the study and/or intervention?
- What was the reason to intervene or what was the problem addressed?
- What was the behavioural level addressed (e.g., individual, group)?
- Which interventions were employed?
- What types of methods were employed to study the mechanism?
- What was the effect and effect size of the intervention?
- Was the study of fishers observational or was it a controlled experiment?
- What was the status of the relationship between the managing body and the fishers (e.g., good/trust, indifferent, bad/distrust)?
- What were the social, economic and environmental outcomes of the studies?
- Were there any unintended effects identified, and if so, which?
- Were any recommendations for future management and policy given?
- Does the study consider the ethics of the research and policy actions, and if so, how?

## Methods

### Search strategy

Drawing upon other review protocols (e.g., [6, 7]), we created a comprehensive list of potential databases and specialised websites. These were listed within the questionnaire (see page 17, S2 File) and stakeholders were asked for feedback. Additionally, the practicality and feasibility of the number of databases and specificity of the keywords were explored by ‘test-driving’ selected keywords. The feedback received from the stakeholder questionnaire and this test-driving process revealed that the amount of literature would be too large to be feasibly addressed systematically. It was therefore decided to focus on three databases: Web of Science (Core Collection), ProQuest (sub-selection of Social Science Core Collection), and EconLit (Table 2). The reason for this selection was the high quality of literature that these three databases return and because of the breath of coverage which is ensured by using a general (Web of Science) and two more research field specific databases (ProQuest for social sciences and EconLit for economics). The three databases will be searched with the keywords presented in Table 3. For each of the databases a specific Boolean search string was constructed from these keywords, for details of these please refer to S3 File. The keywords within the Boolean search string were the results of a pre-scoping exercise on relevant behavioural-economics mechanisms conducted by the lead authors and the BE task group (see “Stakeholder Engagement” section and S3 Table). The keywords were further expanded and commented on through the stakeholder questionnaire before being assessed for practicality (e.g., ambiguity, number of retrieved results in Web of Science).

**Table 2:** Databases and search engines to be used for the systematic review.

Web-based database	Web link
Web of Science (Core Collection)	www.webofknowledge.com
ProQuest–Social Science Core Collection	www.proquest.com
• Education Collection	
• International Bibliography of the Social Sciences (IBSS)	
• Politics Collection	
• Social Science Database	
• Sociology Collection	
EconLit	www.ebsco.com/products/research-databases/econlit

**Table 3:** Keyword groups and function of these.

Keywords	Function
behavio* AND fisher*	Specifies broad field and subjects
human OR soci*	Specifies study area/field
interve* OR experiment* OR mechanis* OR observ* OR chang*	Specifies that we are specifically looking at interventions or observations of behavioural change. Observations were included in order to capture studies which looked at changes as a response to natural or third party interventions.
anchoring OR arbitrage OR (being watched) OR (behavio*econom*) OR bias OR (bounded rationality) OR (co*creation) OR (cognitive bias*) OR (collaborativemanag*) OR (co*management) ORcomplian OR (co*operat*) OR (crowding out) OR (cultural norm*) OR decoy OR default OR (disposition effect) OR (cognitive dissonance) OR labelling OReducat* OR empower OR fairness OR framing OR (game theory) OR heuristic OR (hyperbolic discounting) OR identity ORincentiv* OR (intrinsic motivation*) OR leadership OR (loss aversion) OR management OR (mental accounting) OR mechanism* OR (msccert*) ORnudg* OR (participatorymanag*) OR preference OR (present bias) OR priming OR (probability weighting) OR (procedural utility) OR (prospect theory) OR (satisfaction delay) OR (self*perception) OR (self*serving) OR (self*theory) OR (soci*categor*) OR (soci* learning) OR (social norm*) OR (social proof) OR (soci* factor*) OR (soci* driver*) OR status-quo OR (sunk cost) OR trust OR values	Specifies specific scope relevant to the research question

In addition to extracting studies from web-based databases, stakeholders were given the opportunity to suggest peer-reviewed literature themselves (see stakeholder questionnaire: S2 File and S5 Table).

The search will be conducted in English only. The authors acknowledge that this means that valuable literature and research conducted in other languages will be missed. Being unable to include such literature can cause geographical biases and lack of important different perspectives or elements in the study [8] which result in creating barriers when trying to transfer knowledge internationally [9]. The implied limitations will be acknowledged in the manuscript by citing associated literature such as work by Amano et al. [8], who estimated that up to 35% of relevant literature may be missed if focusing on English language only (in their case in a biodiversity conservation context).

The comprehensiveness of the search was tested by applying the Boolean search string and matching the results with a list of 20 benchmark articles (S4 Table) (as suggested by authors such as Livoreil et al. [10]). The list of benchmark articles was drawn from the references given by Andrews et al. [5] and Battista et al. [4]–two highly relevant and related review studies. From these benchmark articles, AMW made an initial selection of 51 relevant articles, who screened the titles for relevance to the primary research question of this study. AMW, JKH and AS then separately screened the selected articles for relevance using abstracts as well as titles and each selected and ranked the 20 articles they deemed most relevant. From the 51 pre-selected articles, five articles were ranked among the 20 most relevant articles by all three lead authors, 12 by two lead authors and 18 articles were selected by only one lead author. Based on this ranking, the 20 overall highest scoring articles were selected to be used for this benchmark list.

Using this benchmark list, we found that the comprehensiveness of the search strategy (i.e. the number of benchmark articles that our search string retrieves on Web of Science) is only 40%. This appears to be a low number, but during the testing of the search string the lead authors determined that if the Boolean search string was adapted to capture more benchmark articles, the overall search would return a high amount of non-eligible articles. These articles would need to be manually screened and excluded during title, abstract and full-text screening, and the number of eligible articles would not be feasible to include in the systematic review. The lead authors propose that this issue could be caused by the complexity and diversity of (and occasional imprecision in) the terminology related to the field, as well as the accuracy of the indexing of the database [11]. Often, a combination of databases is recommended to ensure coverage of the search, with the Ebsco (of which EconLit is a part) and social-science databases such as PsychINFO being among those which seem to supplement more general ones such as the Web of Science Core Collection [11]. The lead authors have also followed the recommendation to consult experts in the field and ask for specific literature suggestions [12]. We therefore acknowledge that, despite best efforts, the performance of the Boolean search string (S4 Table) indicates that only approximately 40% of articles that were deemed relevant by experts and a qualitative review of the literature will be captured by this search.

### Article screening

We will follow the ROSES flow diagram during article screening, applying the consistency checks and eligibility criteria [13]. All articles returned by the search procedure will be divided between AMW and JKH, who will conduct title and abstract screening of the articles and decide on their inclusion or exclusion. Articles that pass through to full-text screening will be assessed for eligibility based on the entire article content. Articles that fulfill the eligibility criteria will then be reviewed using the synthesis form (S4 File). Articles will be assigned across the lead authors and to volunteers from the BE task team as well as Mary Mackay. First, however, two articles will be selected at random and all reviewers will independently review these articles according to the inclusion criteria below and populate the synthesis form independently. The authors will then meet to discuss and compare their decisions and the collated information by each author and check for consistency before the articles are assigned. This exercise will be repeated once all the reviewers have screened several papers.

### Eligibility criteria

#### Eligible populations or subjects

Studies that focus on the behaviour of marine fishers (i.e., not in freshwater rivers or lakes) will be considered. We exclude freshwater fisheries because they have several differences that make comparisons difficult (e.g., regulation, access, scale, and markets). Fishers of any scale (recreational, artisanal, small-scale, industrial) or any sector (pelagic, demersal, shellfish, shrimp, mixed), operating in any region (local, regional, national, international, offshore) anywhere in the world will be considered. Although they may operate in similar environmental contexts, we exclude other marine activities such as aquaculture, gleaning or land-based harvests of marine products (e.g., seaweed) because they tend to have separate regulatory and institutional contexts and often occur in semi-privatised spaces.

#### Eligible interventions

If interventions are studied, any interventions that investigate fisher behaviour/decision-making and which do not solely relate to traditional economic decision-making (e.g., location choice to maximize profits) will be considered. The interventions can be instigated by any relevant actors (e.g., scientists, policy makers, NGOs, or fishers themselves).

#### Eligible outcomes

Any behavioural change in common practice which may have been an effect of an intervention, as well as those interventions which showed no effect on behaviour (i.e., negative results that have been published will also be considered eligible) will be considered.

#### Eligible type of studies

We will only include studies that directly involve marine fishers and which have a field or experimental component. We consequently will not include articles which are based on theoretical models or those which extrapolate findings from other populations to fishers. Peer-reviewed articles published at any point in time will be considered. The only limitation in regard to the timeframe of the study is how far each database goes back in time. For the three chosen databases, being Web of Science (Core Collection), ProQuest (sub-selection of Social Science Core Collection), and EconLit this is 1945, 1914 and 1889, respectively. A list of all excluded articles will be provided in the supplementary information of the review article. For articles excluded at either the abstract and full text screening level, reason(s) for exclusion will also be recorded.

### Study validity assessment

The aim of the review study is to provide a narrative overview, based on a systematic review of the literature, of behavioural-economics mechanisms that drive fisher behavior. We are relying on peer-reviewed publications. As the work has already been peer reviewed in the publication process, we do not aim to appraise individual studies or the validity of the body of evidence ourselves.

### Data extraction

Information on each of the studies will be collected via a password-protected form (S4 File) made available to all text-screening authors through the General Data Protection Regulation (GDPR)—compliant UK online survey platform (www.onlinesurveys.ac.uk) [14]. The form collects the following information, if available, for each article under review:

**Meta-data extraction**.

- Name of screening author
- Article doi
- Article title
- Country(ies) where study is based
- Continent(s) where study is based
- Major ocean basin(s) the study is concerned with
- Geographic scale (e.g., local, regional, …)
- Fisheries scale (e.g., recreational, artisanal, small-scale, …)
- Gear type employed in fisheries the study is concerned with (e.g., longlines, purse seine, pole and line, …)
- Fisheries commercial group (e.g., flatfishes, anchovies, cod-forms, …)
- Whether the study is concerned with particularly depleted fish stock(s) (e.g., Bluefin tuna, Pacific bonito, European sea sprat, …)
- Instigating party of the study (e.g., scientist, policy maker, fishers, …)

**Data extraction**.

- Type of behavioural factor found to influence fisher behaviour in the study (after Andrews et al. [5])
- Behavioural-economics mechanism(s) employed in study (e.g., priming, framing, decoy-effect, …)
- Why the study or intervention was set up or what the investigated problem was
- What type of method was employed in the study (e.g., experiment–lab, experiment–field, descriptive–qualitative, …)
- Behavioural level of intervention and/or observation (e.g., individuals, groups, groups and individuals)
- Comments on methodology
- Description of intervention
- Description of effect and effect size (if applicable)
- Relevance to fisheries (e.g., study in real-world context, not *in situ* but related to, and involving fishers, not *in situ* nor involving fishers but related to fishers, not directly related to fisheries but theoretically applicable)
- Relationship between fishers and managers (e.g., good/trust, indifferent, bad/distrust)
- Description of overall outcome
- Whether social, economic or environmental outcomes were considered and if so how
- Whether any unintended effects were noted and if so which
- Whether any management strategies and policy recommendations were given and if so which
- Whether ethics were considered (including but not limited to formal ethics approcal, an ethics statement, or the mention of the ethical considerations of applying insights in a management context)
- One-sentence conclusion of the study

### Data synthesis

**Narrative synthesis**.

- An overview table of behavioural-economics mechanisms and their evidence base is a key expected output of this study. An initial inventory of behavioural mechanisms (drawn from a pre-scoping exercise with stakeholders) was used to design the search terms and synthesis form. New mechanisms will be added to the full-text synthesis form in regular intervals until conceptual saturation is reached for the overall inventory (i.e., when no new patterns or themes emerge, as in Morse [15]). A table in the publication will then outline the mechanisms, their definitions, examples from the reviewed literature, the geographic scale, fisheries scale, effect size, environmental outcome and general outcome linked to the mechanism. The aim of this synthesis table is to communicate insights into the key mechanisms and provide an overview of where and how they have been employed.
- Figures (such as bar charts) will be used to visually represent synthesised information about the literature such as geographic scale, fisheries scale, commercial fisheries group, applied method type, behavioural factor type, behavioural level, relationship type between fishers and managers and whether ethical considerations were taken into account. While we acknowledge that human behaviour is often context-dependent, for such a large synthesis we will not have scope to consider the regulatory, cultural, economic and institutional context for each included study.
- Information on the reviewed literature’s location (continent, country and major ocean basin) will be used to create a geographic heat map of where studies have been conducted. This will help evaluate whether there is any bias towards particular countries or regions.
- Meta-data on the fishing gear type, commercial fisheries group and whether the study is concerned with a particularly depleted fishing stock will be used to investigate whether behavioural-economics mechanisms may be under-utilised in particular fisheries.

#### Qualitative synthesis

The narrative synthesis and descriptive statistics will be supported by qualitative analysis of why an intervention may have been set up, what problem the studies seek to address as well as any future management and policy recommendations which may have been provided in the literature. Considering the instigating party of each study will help gain a better understanding of who currently has an interest in employing behavioural-economics mechanisms in a fisheries context. Finally, a qualitative analysis of any ethical considerations made in the literature will provide an overview of what ethical aspects of behavioural economics interventions were considered and how they may have been addressed. For the latter we will consider dedicated ethics statements as well as any ethical considerations described within the study which may include subtle reflections on the ethical implications of altering fisher behavior (e.g. in the discussion section). The aim of this is to get a first indication of the general perception of the ethics of employing behavioural economic in the context of fisheries management.

### Risk of publication bias

The authors have no conflict of interest in the nature of the results. The lead authors are a heterogeneous group and have consulted multiple stakeholders to define the purpose of the review. There is no risk of publication bias, beyond the language bias mentioned previously, because this is simply an overview of past research on behavioural- economics mechanisms in fisheries contexts.

### Knowledge gap and cluster identification

Together with the stakeholders the lead authors have designed the review to target knowledge clusters and knowledge gaps with the aim of synthesising them. Descriptive statistics will be used for the data synthesis to report the distribution of research in terms of mechanism, method, scale, sector, etc. in order to provide a descriptive impression of the knowledge clusters.

### Procedural independence

Most of the stakeholders work in the field of fisheries social science, and the lead authors have incorporated much of their feedback in designing this protocol. It is a varied group in terms of discipline and region, and all were given equal opportunity to provide written feedback on the study design. It is inevitable that some of the published work of the stakeholders (and co-authors of this work) will be retrieved as part of the review, but the reviewers will process and review each of the papers according to the protocol described above. Currently, none of the lead authors have published work that will be included in the review. However, AS has an article accepted which may be included in the final search. The lead authors have asked the stakeholders of the study for literature suggestions which will be added to the review, some of which may not be retrieved by the search terms. This may introduce a bias. For transparency, we will include the list of the literature that was suggested by the stakeholders in the supplementary information of the manuscript and highlight which of these were and were not captured by the systematic review search.

### Ethics and dissemination

This systematic review protocol has been written following ROSES *pro forma* reporting guidelines [13] (for completed ROSES checklist refer to S1 Table). This systematic review study is on peer-reviewed literature and we therefore will not obtain ethics clearance or documentation. The individuals noted in the supplementary material of this manuscript have given written informed consent (as outlined in PLOS consent form) to publish these case details. We expect to publish the systematic review in a peer-reviewed journal.

## Discussion

This study responds to an important request by the BE task group of ICES WGMARS, who have identified the need for a comprehensive overview of studies which currently employ behavioural-economics mechanisms in a fisheries-management context. This registered report protocol was developed together with a large group of stakeholders (S2 Table) and is specifically tailored to their needs. The narrative and qualitative analyses which will be conducted will provide insights into behavioural-economics mechanisms in a fisheries context for these and other relevant stakeholders and decision makers in fisheries management. More specifically, the review will consider geographic location, scale, fisheries sector, initiating party, ethics considerations and whether any unintended effects were observed when employing behavioural-economics mechanisms, contributing a novel synthesis of the peer-reviewed work to date. Any policy and management recommendations given in these studies will also be presented and the overall insights will be used to discuss the future direction and potential use of behavioural economics in a fisheries-management context. The study’s limitations include the restriction to the English language and the exclusion of non-peer reviewed studies and reports. However, we believe that these omissions are justified for the sake of feasibility and rigour. Another limitation, revealed by the benchmark article screening, is that not all relevant and prominent studies into behavioural economics in a fisheries context were captured using the Boolean search term. We therefore acknowledge that the review will not be a fully comprehensive review of all work published on the subject, but will rather offer a first overview of the field. By providing an overview of mechanisms, their effects, and relevant information about the state of research on this topic so far, this systematic review will be a useful tool for fisheries-management stakeholders. Many of the stakeholders who are likely to employ insights in the future helped shape the review protocol. We are therefore confident that the product of this review will provide a significant contribution to the field.

## Supporting information

S1 Table Filled-in ROSES checklist and meta-data form. (XLSX)

S2 Table Overview of WGMARS stakeholder members and roles. (DOCX)

S3 Table Behavioural-economics mechanism table. (DOCX)

S4 Table Benchmark articles present in search. (DOCX)

S5 Table Peer-reviewed articles suggested by WGMARS stakeholders. (DOCX)

S1 File Stakeholder webinar. (PDF)

S2 File Stakeholder questionnaire. (PDF)

S3 File Details of boolean search string for each databases. (PDF)

S4 File Synthesis form. (PDF)

## References

[1] Fulton EA, Smith ADM, Smith DC, Putten IE van. Human behaviour: the key source of uncertainty in fisheries management. Fish and Fisheries. 2011;12: 2–17. doi: 10.1111/j.1467-2979.2010.00371.x

[2] ICES. 2020. ICES Working Group on Maritime Systems (WGMARS). ICES Scientific Reports. 2:63. 22 pp. doi: 10.17895/ICES.PUB.6104

[3] Eikeset AM, Richter AP, Diekert FK, Dankel DJ, Stenseth NChr. Unintended consequences sneak in the back door: making wise use of regulations in fisheries management. In: Belgrano A, Fowler CW, editors. Ecosystem Based Management for Marine Fisheries: An Evolving Perspective. Cambridge: Cambridge University Press; 2011. pp. 183–217. doi: 10.1017/CBO9780511973956.009

[4] Battista W, Romero-Canyas R, Smith SL, Fraire J, Effron M, Larson-Konar D, et al. Behavior Change Interventions to Reduce Illegal Fishing. Frontiers in Marine Science. 2018;5. doi: 10.3389/fmars.2018.00403

[5] Andrews EJ, Pittman J, Armitage DR. Fisher behaviour in coastal and marine fisheries. Fish and Fisheries. 2020;00: 1–14. doi: 10.1111/faf.12529

[6] Hughes KM, Kaiser MJ, Jennings S, McConnaughey RA, Pitcher R, Hilborn R, et al. Investigating the effects of mobile bottom fishing on benthic biota: a systematic review protocol. Environmental Evidence. 2014;3: 23. doi: 10.1186/2047-2382-3-23 PMC11476316 39402692

[7] Ward-Campbell BMS, Valere B. What are the impacts of small-scale dredging activities on inland fisheries productivity? A systematic review protocol. Environmental Evidence. 2018;7: 9. doi: 10.1186/s13750-018-0119-1

[8] Amano T, González-Varo JP, Sutherland WJ. Languages Are Still a Major Barrier to Global Science. PLOS Biology. 2016;14: e2000933. doi: 10.1371/journal.pbio.2000933 28033326 PMC5199034

[9] Meneghini R, Packer AL. Is there science beyond English? Initiatives to increase the quality and visibility of non-English publications might help to break down language barriers in scientific communication. European Molecular Biology Organization Reports. 2007;8: 112–116. doi: 10.1038/sj.embor.7400906 17268499 PMC1796769

[10] Livoreil B, Glanville J, Haddaway NR, Bayliss H, Bethel A, de Lachapelle FF, et al. Systematic searching for environmental evidence using multiple tools and sources. Environmental Evidence. 2017;6: 23. doi: 10.1186/s13750-017-0099-6

[11] Bramer WM, Rethlefsen ML, Kleijnen J, Franco OH. Optimal database combinations for literature searches in systematic reviews: a prospective exploratory study. Systematic Reviews. 2017;6: 245. doi: 10.1186/s13643-017-0644-y 29208034 PMC5718002

[12] Stevinson C, Lawlor DA. Searching multiple databases for systematic reviews: added value or diminishing returns? Complementary Therapies in Medicine. 2004;12: 228–232. doi: 10.1016/j.ctim.2004.09.003 15649836

[13] Haddaway NR, Macura B, Whaley P, Pullin AS. ROSES RepOrting standards for Systematic Evidence Syntheses: pro forma, flow-diagram and descriptive summary of the plan and conduct of environmental systematic reviews and systematic maps. Environmental Evidence. 2018;7: 7. doi: 10.1186/s13750-018-0121-7

[14] EU General Data Protection Regulation (GDPR): Regulation (EU) 2016/679 of the European Parliament and of the Council of 27 April 2016 on the protection of natural persons with regard to the processing of personal data and on the free movement of such data, and repealing Directive 95/46/EC (General Data Protection Regulation), OJ 2016 L 119/1.

[15] Morse JM. The Significance of Saturation. Qual Health Res. 1995;5: 147–149. doi: 10.1177/104973239500500201

