# Supplementary material for: Behavioural economics in fisheries: A systematic review protocol
Source: PLoS One. 2021 Aug 26;16(8):e0255333. doi: 10.1371/journal.pone.0255333 (PMC8389455; doi:10.1371/journal.pone.0255333)
Supplement: S2 Table — (DOCX) [file pone.0255333.s002.docx]

**S2 Table:** List of stakeholders and their role in the study

| **Name** | **Country** | **Stakeholder Role** |
| --- | --- | --- |
| Alina Wieczorek | Ireland | Lead author |
| Amanda Schadeberg | Netherlands | Lead author |
| Andries Richter | Netherlands | BE-task group |
| Debbi Pedreschi | Ireland | BE-task group |
| Dorothy Dankel | Norway | BE-task group |
| Ingrid van Putten | Australia | BE-task group |
| Julie Krogh Hallin | Denmark | Lead author |
| Leyre Goti | Germany | BE-task group |
| Patricia M. Clay | United States | BE-task group |
| Rolf Groeneveld | Netherlands | BE-task group |
| Sarah B.M. Kraak | Germany | BE-task group |
| [WGMARS members](https://www.ices.dk/community/groups/Pages/Members.aspx?Acronym=WGMARS) as listed here: <https://www.ices.dk/community/groups/Pages/Members.aspx?Acronym=WGMARS> (accessed 3^rd^ June 2021) | - | WGMARS |
