# Supplementary material for: Behavioural economics in fisheries: A systematic review protocol
Source: PLoS One. 2021 Aug 26;16(8):e0255333. doi: 10.1371/journal.pone.0255333 (PMC8389455; doi:10.1371/journal.pone.0255333)
Supplement: S3 Table — (DOCX) [file pone.0255333.s003.docx]

**S3 Table:** Behavioural Economics Mechanism Table

* Examples marked with an asterisk are intended to assist with the systematic review protocol and are not reflections of published literature or even the opinions of the authors. Rather, they are propositions intended to clarify the mechanism and its definition.

| **Grouping idea** | **Mechanism/Driver** | **Definition/explanation** | **Example in fisheries** |
| --- | --- | --- | --- |
| **Cognitive bias/ Psychology** | **Reference dependence/Shifting baselines** | The tendency for a reference or baseline to set the point against which changes are seen as ‘gains’ or ‘losses’. Changing the reference point changes whether people perceive the change as a win or loss. | If North Sea cod is assessed only since 2000, it appears to be flourishing. Moving the baseline to 1970 reveals an overall decline (loosely interpreted from Pauly [1] on shifting baselines) which can affect behaviour. |
|  | **Anchoring effect** | The tendency to be heavily influenced by the initial piece of information offered when making decisions, leading to insufficient adjustment to new information [2,3]. | If a preliminary TAC estimate is publicised, fishers and governments are likely to hold onto that initial number and have trouble adjusting to the updated figure, no matter whether it’s higher or lower.* |
|  | **Non-linear probability weighting** | The tendency to overestimate low probability events and underestimate high probability ones. | Workers in fisheries sector are less risk averse than the workers in other occupations and may have gotten used to a risky work environment [4]. |
|  | **Self-image** | The tendency for one’s environmental self-identity to relate to one's obligation-based intrinsic motivation (that is, feelings of moral obligation) to act pro-environmentally, which in turn affects pro-environmental actions [5]. | Influencing individual self-identities to align with sustainability can create conditions that incentivize a company, country, or individual to fish sustainably, curb illegal fishing, or create large marine reserves as steps to enhance reputation or self-image [5]. |
|  | **Priming** | The tendency to, when exposed to an external stimulus (e.g., a list of words describing old people), activate a mental construct associated with this stimulus (e.g., “being old”), which may in turn affect subsequent behaviour without one necessarily being aware of this influence. | Fishers misreported coin tosses to their economic advantage more strongly in a treatment where they were primed with the EU logo [6,7]. |
|  | **Framing** | The tendency to decide on options based on whether the options are presented with positive or negative connotations (e.g., as a loss or as a gain). There is a related tendency to avoid risk when a positive frame is presented, but seek risks when a negative frame is presented. | Framing economic incentives differently can have different effects on fishing extraction and earnings [8].  Note: This differs from priming in that it is a constant process (not just a single ‘priming moment’). |
|  | **Hedonic Framing** | The tendency to split a gain into smaller gains to maximize the pleasure, but combine losses into a single loss to minimize pain. In other words, two individual gains are perceived to be more valuable than a single larger gain of the same value. Similarly, one big loss is easier to bear than two small ones [9]. | Following this theory, it would be expected that opening restricted fishing grounds for one week at a time twice a season would be more supported by fishers than opening for a single period of two weeks.* |
|  | **Paradox of Choice** | The tendency for more options or choices to make a decision more difficult to reach, potentially hurting well-being in the process. Popularized by psychologist Barry Schwartz [9], the theory has been tested and analysed in many different ways over the years [10]. | It is easier to choose from 3 options than 30. * |
|  | **Ego depletion** | The tendency for the capacity to make good decisions to be inhibited by both decision overload and external fatigue. | The practical reality of being at sea leads to extreme fatigue and fishers can avoid making decisions. To change behaviour, the moment of decision needs to be moved to when they are rested.* |
|  | **Effect of being watched / Panopticism** | The tendency for compliant behaviour to increase when one is aware that they are being watched, even if it is not known when or by whom [11]. In other words, the possibility of being watched makes people behave *as if* they are being watched (whether they are watched or not). | Vessel monitoring systems, Automatic Identification System, drones, control vessels, cameras on-board to monitor catches, etc. [6]. |
|  | **Status-quo bias** | The tendency to prefer things to stay the same by doing nothing or by sticking with a decision made previously (Samuelson, & Zeckhauser, 1988). This may happen even when only small transition costs are involved and the importance of the decision is great [12]. | A group of shrimp fishers are offered an alternative way of commercialisation that would be more profitable and more resilient to ecological shocks, but they nevertheless prefer the status quo. ^*^ |
|  | **Decoy effect** | The tendency to accept paying a higher price when a ‘bad choice’ is offered in the list of options, making the others looks more attractive or reasonable. | The Economist offered subscriptions with the options a) ‘print & web’ for $125 and b) ‘web only’ for $59. Most people chose web only. When they introduced a third option (a decoy) c) ‘print only’ for $125 most people chose a) print & web ($125) because it seemed like the better deal compared to print only for the same price. The introduction of the unattractive ‘decoy’ option of print-only prompted more people to take the more expensive option [13]. |
|  | **Availability heuristic** | The tendency to overestimate the importance and likelihood of events given the greater availability of information. | The tendency for fishers to attribute their hardships to marine space allocation to windmills because opposition to windmills is a highly salient discourse in their networks compared to, for example, declining catches caused by climate change-driven species shifts. * |
|  | **Belief bias** | The tendency to base the strength of an argument on the believability or plausibility of its conclusion. | Beam trawl fishers may rely on an analogy between fishing and farming to argue that bottom trawling is good for the seabed because it ‘ploughs’ the bottom and encourages new growth. |
|  | **Confirmation bias** | The tendency to focus only on information that confirms existing preconceptions. | Young et al. [14] identify the tendency for fisheries management science to rely on a small number of measures (e.g. ITQs) as panaceas. |
|  | **Gambler’s fallacy** | The tendency to believe that future probabilities are altered by past events when in fact they are unchanged. These can be positive or negative assumptions. | “I’ve had three bad weeks fishing, so I’m bound to have a good catch this week”.* |
|  | **Illusion of validity** | The tendency to overestimate one’s ability to make accurate predictions, especially when the data appears to tell a coherent ‘story'. | Fisheries managers overestimating the certainty of their models and predictions [15,16]. |
|  | **Ostrich effect** | The tendency to avoid negative information by pretending it doesn't exist. | Wilful ignorance is a constitutive feature of policy processes [18]. |
|  | **Post-purchase rationalization** | The tendency to retrospectively ascribe positive attributes to an option one has selected. | Retroactively justifying ITQs as the best option because they were the option that was implemented (see arguments in Bromley [17]). |
|  | **Risk compensation** | The tendency to take bigger risks when perceived safety increases, and the tendency to be more careful when perceived risk increases. | “Now that we’ve got the new equipment, we can cut the time spent on maintenance” or “The weather is calm today, I don’t have to wear my life jacket”. * |
|  | **Clustering illusion** | The tendency to incorrectly overestimate the importance of small patterns (in data). Data here can also be mental data. | “Last week I had a catch full of rays so they must be doing well.”* |
|  | **Informational influence** | The tendency for new information or arguments provided in a group discussion to change a group member’s attitudes, beliefs, or behavior. Informational influence is likely to be stronger when a person is uncertain about the correct interpretation of reality and/or the correct behavior in a given context and therefore looks to other group members for guidance [18]. | Members of co-operatives or producer organisations will tend to follow the majority opinion on issues that they personally are not informed about.* |
| **Loss aversion**  “Losses loom larger than gains” (Kahneman & Tversky, [19]). It is thought that the pain of losing is psychologically about twice as powerful as the pleasure of gaining [12]. | **Endowment Effect** | The tendency for individuals to value an owned object higher, often irrationally, than its market value [21]. | A fisher prefers to do a costly renovation of the vessel he inherited from his father than purchase a newer more cost-effective vessel.* |
|  | **Sunk cost bias** | The tendency to continue a behaviour or endeavour as a result of previously invested resources (time, money or effort) (Arkes & Blumer [20]). This fallacy can also be viewed as bias resulting from an ongoing commitment [12]. | Because of the money and time already invested, a fisher prefers to continue to use a specific technique even if it is no longer profitable.* |
|  | **Disposition effect** | The tendency to be reluctant to sell assets that have lost value and the invers- related greater likelihood of selling assets that have made gains (Shefrin & Statman [21]) [12]. | A fisher’s reluctance to sell quota for a lower price than the one he purchased it for, or inversely the fisher’s increased likelihood of selling quota when its value increases, even if holding it longer would be more profitable in the long term.* |
|  | **Present bias** | The tendency to prefer settling for a smaller present reward than to wait for a larger future reward, in a trade-off situation. It describes the trend of overvaluing immediate rewards, while putting less worth in long-term consequences. | If managers continually ‘reset’ their commitment to harvesting strategies, bowing to pressure to choose short-term rewards (i.e. higher catches) at the expense of long-term benefits (i.e. stock preservation) then the optimal policy can appear to be to harvest the stock to extinction because they are biased towards the returns In the present. [22] |
|  | **Hyperbolic discounting** | The tendency for people to increasingly choose a smaller sooner reward over a larger-later reward as the delay occurs sooner rather than later in time [25]. | The same effect as above, but it becomes more extreme the further into the future the large benefit is (i.e., it is easier to implement restrictions for the sake of a pay-off in five years than in fifty years).* |
| **Social Norms**  Social norms signal appropriate behaviour and are classed as behavioural expectations or rules within a group of people (Dolan et al. [23]) [12]. | **Injunctive / Social norms / Social license to operate** | The tendency to want to comply with social norms. One can influence behaviour by emphasizing injunctive social norms: (i.e., how one should behave). | A poster campaign that states ‘a real fisherman releases sharks immediately’.*  MSC Certification sets standard for socially acceptable fishing and gives those who have the label have been given a so-called ‘social license to operate’ [24]. |
|  | **Descriptive norms** | The tendency to want to comply with social norms. One can influence behaviour by invoking descriptive social norms (i.e., how the majority behaves). | A poster campaign that states ‘most fisherman choose to release sharks immediately, before processing their catch’’* Mackay et al. (2019), in a laboratory experiment, successfully used a descriptive social norm to nudge recreational fishers to stick to bag limit. [25] |
|  | **Bandwagon effect** | The tendency for the uptake of beliefs and ideas to increase the more that they have already been adopted by others. | The idea of critical mass in a transition (for better or for worse outcomes). E.g., “Everybody else is using beam trawls so we should switch, too.”* |
|  | **Blind spot bias** | The tendency to view oneself as less biased than others. | Fisheries scientists see their work as apolitical, but the inputs of fishers or other stakeholders as biased.* |
|  | **Courtesy bias** | The tendency to give an opinion or conclusion that is viewed as more socially acceptable so as to avoid causing offence or controversy. | Fishers do not feel comfortable talking about the rationale behind their discarding practices when in the presence of someone they perceive as being motivated by conservation, because discarding is usually seen as a destructive practice.* |
|  | **Reactive devaluation** | The tendency to devalue an idea because it originated from an adversary or opponent. | If a conservation eNGO advocates for surveillance cameras on board vessels, fishers will automatically oppose it; but if the suggestion comes from within the co-operative or producer organization then they may support it.* |
|  | **Stereotyping** | The tendency to assume that a person has characteristics because they are a member of a group. | “Fishers never listen to managers.”* |

**References**

1. Pauly D. Anecdotes and the shifting baseline syndrome of fisheries. Trends Ecol Evol. 1995;10: 430. doi:10.1016/s0169-5347(00)89171-5

2. Tversky A, Kahneman D. Judgment under Uncertainty: Heuristics and Biases. Science. 1974;185: 1124–1131. doi:10.1126/science.185.4157.1124

3. Furnham A, Boo HC. A literature review of the anchoring effect. The Journal of Socio-Economics. 2011;40: 35–42. doi:10.1016/j.socec.2010.10.008

4. Nguyen Q, Leung P. Do Fishermen Have Different Attitudes Toward Risk? An Application of Prospect Theory to the Study of Vietnamese Fishermen. J Agric Resour Econ. 2009;34: 518–538.

5. van der Werff E, Steg L, Keizer K. It is a moral issue: The relationship between environmental self-identity, obligation-based intrinsic motivation and pro-environmental behaviour. Global Environmental Change. 2013;23: 1258–1265. doi:10.1016/j.gloenvcha.2013.07.018

6. Kraak SBM, Hart PJB. Creating a Breeding Ground for Compliance and Honest Reporting Under the Landing Obligation: Insights from Behavioural Science. In: Uhlmann SS, Ulrich C, Kennelly SJ, editors. The European Landing Obligation: Reducing Discards in Complex, Multi-Species and Multi-Jurisdictional Fisheries. Cham: Springer International Publishing; 2019. pp. 219–236. doi:10.1007/978-3-030-03308-8_11

7. Drupp MA, Khadjavi M, Quaas MF. Truth-telling and the regulator. Experimental evidence from commercial fishermen. European Economic Review. 2019;120: 103310. doi:10.1016/j.euroecorev.2019.103310

8. Ertör-Akyazi P. Formal versus informal institutions: Extraction and earnings in framed field experiments with small-scale fishing communities in Turkey. Marine Policy. 2019;109: 103673. doi:10.1016/j.marpol.2019.103673

9. Schwartz B. The paradox of choice: Why more is less. New York, NY, US: HarperCollins Publishers; 2004. pp. xi, 265.

10. www.zachhellermarketing.com.

11. Foucault M. Discipline and Punish: The Birth of the Prison. Vintage Books; 1995.

12. www.behavioraleconomics.com.

13. The Predictably Irrational by Dan Ariely – Audiobooks on Google Play. [cited 11 Mar 2021]. Available: https://play.google.com/store/audiobooks/details/The_Predictably_Irrational?id=AQAAAAAUxnvGfM&hl=en_GB&gl=IE

14. Young OR, Webster DG, Cox ME, Raakjær J, Blaxekjær LØ, Einarsson N, et al. Moving beyond panaceas in fisheries governance. PNAS. 2018;115: 9065–9073. doi:10.1073/pnas.1716545115

15. Dankel DJ, Aps R, Padda G, Röckmann C, van der Sluijs JP, Wilson DC, et al. Advice under uncertainty in the marine system. ICES Journal of Marine Science. 2012;69: 3–7. doi:10.1093/icesjms/fsr179

16. Rochet M-J, Rice JC. Simulation-based management strategy evaluation: ignorance disguised as mathematics? ICES Journal of Marine Science. 2009;66: 754–762. doi:10.1093/icesjms/fsp023

17. Bromley DW. Abdicating Responsibility: The Deceits of Fisheries Policy. Fisheries. 2009;34: 280–290. doi:https://doi.org/10.1577/1548-8446-34.6.280

18. www.psychology.iresearchnet.com.

19. Kahneman D, Tversky A. Prospect theory: An analysis of decision under risk. New York, NY, US: Cambridge University Press; 1988. p. 214. doi:10.1017/CBO9780511609220.014

20. Arkes HR, Blumer C. The psychology of sunk cost. Organizational Behavior and Human Decision Processes. 1985;35: 124–140. doi:10.1016/0749-5978(85)90049-4

21. Shefrin H, Statman M. The Disposition to Sell Winners Too Early and Ride Losers Too Long: Theory and Evidence. The Journal of Finance. 1985;40: 777–790. doi:10.2307/2327802

22. Duncan S, Hepburn C, Papachristodoulou A. Optimal harvesting of fish stocks under a time-varying discount rate. J Theor Biol. 2011;269: 166–173. doi:10.1016/j.jtbi.2010.10.002

23. Dolan P, Hallsworth M, Halpern D, King D, Vlaev I. MINDSPACE: influencing behaviour for public policy. London, UK: Institute of Government; 2010 [cited 17 Mar 2021]. Available: http://www.instituteforgovernment.org.uk/publications/

24. Putten I van, Longo C, Arton A, Watson M, Anderson CM, Himes-Cornell A, et al. Shifting focus: The impacts of sustainable seafood certification. PLOS ONE. 2020;15: e0233237. doi:10.1371/journal.pone.0233237

25. Mackay M, Yamazaki S, Jennings S, Sibly H, Putten I, Putten I, et al. The influence of nudges on compliance behaviour in recreational fisheries: a laboratory experiment. 2019. doi:10.1093/ICESJMS/FSZ020
