# Supplementary material for: Behavioural economics in fisheries: A systematic review protocol
Source: PLoS One. 2021 Aug 26;16(8):e0255333. doi: 10.1371/journal.pone.0255333 (PMC8389455; doi:10.1371/journal.pone.0255333)
Supplement: S4 Table — (DOCX) [file pone.0255333.s004.docx]

**S4 Table**: Benchmark articles present in search

| **Benchmark article** | **Present in search** |
| --- | --- |
| Andrews EJ, Pittman J, Armitage DR. Fisher behaviour in coastal and marine fisheries. Fish and Fisheries. n/a. doi:10.1111/faf.12529 | Present |
| Arias A, Sutton S. Understanding Recreational Fishers’ Compliance with No-take Zones in the Great Barrier Reef Marine Park. Ecology and Society. 2013;18. doi:10.5751/ES-05872-180418 | Present |
| Arias A, Cinner J, Jones R, Pressey R. Levels and drivers of fishers’ compliance with marine protected areas. Ecology and Society. 2015;20. doi:10.5751/ES-07999-200419 | Present |
| Battista W, Romero-Canyas R, Smith SL, Fraire J, Effron M, Larson-Konar D, et al. Behavior Change Interventions to Reduce Illegal Fishing. Front Mar Sci. 2018;5. doi:10.3389/fmars.2018.00403 | Present |
| Bergseth BJ, Roscher M. Discerning the culture of compliance through recreational fisher’s perceptions of poaching. Marine Policy. 2018;89: 132–141. doi:10.1016/j.marpol.2017.12.022 | Present |
| Bova CS, Halse SJ, Aswani S, Potts WM. Assessing a social norms approach for improving recreational fisheries compliance. Fisheries Management and Ecology. 2017;24: 117–125. doi:10.1111/fme.12218 | Present |
| Christou M, Haralabous J, Stergiou KI, Damalas D, Maravelias CD. An evaluation of socioeconomic factors that influence fishers’ discard behaviour in the Greek bottom trawl fishery. Fisheries Research. 2017;195: 105–115. doi:10.1016/j.fishres.2017.07.003 | Not present |
| Daw TM, Cinner JE, McClanahan TR, Brown K, Stead SM, Graham NAJ, et al. To Fish or Not to Fish: Factors at Multiple Scales Affecting Artisanal Fishers’ Readiness to Exit a Declining Fishery. PLOS ONE. 2012;7: e31460. doi:10.1371/journal.pone.0031460 | Not present |
| Eliasen SQ, Papadopoulou K-N, Vassilopoulou V, Catchpole TL. Socio-economic and institutional incentives influencing fishers’ behaviour in relation to fishing practices and discard. ICES Journal of Marine Science. 2014;71: 1298–1307. doi:10.1093/icesjms/fst120 | Not present |
| Gallic BL, Cox A. An economic analysis of illegal, unreported and unregulated (IUU) fishing: Key drivers and possible solutions. Marine Policy. 2006;30: 689–695. doi:10.1016/j.marpol.2005.09.008 | Not present |
| Gezelius SS. Do Norms Count? State Regulation and Compliance in a Norwegian Fishing Community. Acta Sociologica. 2002;45: 305–314. | Not present |
| Gutiérrez NL, Hilborn R, Defeo O. Leadership, social capital and incentives promote successful fisheries. Nature. 2011;470: 386–389. doi:10.1038/nature09689 | Not present |
| Hatcher A, Jaffry S, Thébaud O, Bennett E. Normative and Social Influences Affecting Compliance with Fishery Regulations. Land Economics. 2000;76: 448–461. doi:10.2307/3147040 | Not present |
| Hilborn R. Managing fisheries is managing people: what has been learned? Fish and Fisheries. 2007;8: 285–296. doi:10.1111/j.1467-2979.2007.00263_2.x | Not present |
| Hønneland G. Compliance in the Barents Sea fisheries. How fishermen account for conformity with rules. Marine Policy. 2000;24: 11–19. doi:10.1016/S0308-597X(98)00058-X | Not present |
| Karr KA, Fujita R, Carcamo R, Epstein L, Foley JR, Fraire-Cervantes JA, et al. Integrating Science-Based Co-management, Partnerships, Participatory Processes and Stewardship Incentives to Improve the Performance of Small-Scale Fisheries. Front Mar Sci. 2017;4. doi:10.3389/fmars.2017.00345 | Not present |
| Lopez MC, Murphy JJ, Spraggon JM, Stranlund JK. Comparing the Effectiveness of Regulation and Pro-Social Emotions to Enhance Cooperation: Experimental Evidence from Fishing Communities in Colombia. Economic Inquiry. 2012;50: 131–142. doi: 10.1111/j.1465-7295.2010.00344.x | Present |
| Macusi ED, Katikiro RE, Babaran RP. The influence of economic factors in the change of fishing strategies of anchored FAD fishers in the face of declining catch, General Santos City, Philippines. Marine Policy. 2017;78: 98–106. doi:10.1016/j.marpol.2017.01.016 | Present |
| Raakjær Nielsen J, Mathiesen C. Important factors influencing rule compliance in fisheries lessons from Denmark. Marine Policy. 2003;27: 409–416. doi:10.1016/S0308-597X(03)00024-1 | Not present |
| Putten IE van, Kulmala S, Thébaud O, Dowling N, Hamon KG, Hutton T, et al. Theories and behavioural drivers underlying fleet dynamics models. Fish and Fisheries. 2012;13: 216–235. doi:10.1111/j.1467-2979.2011.00430.x | Not present |
