# Supplementary material for: Behavioural economics in fisheries: A systematic review protocol
Source: PLoS One. 2021 Aug 26;16(8):e0255333. doi: 10.1371/journal.pone.0255333 (PMC8389455; doi:10.1371/journal.pone.0255333)
Supplement: S5 Table — (DOCX) [file pone.0255333.s005.docx]

**S5 Table:** Peer-reviewed articles suggested by WGMARS stakeholders

| **Suggested by** | **Reference** |
| --- | --- |
| Patricia M. Clay | Abbott JK, Haynie AC, Reimer MN. Hidden Flexibility: Institutions, Incentives, and the Margins of Selectivity in Fishing. Land Economics. 2015;91: 169–195. doi:10.3368/le.91.1.169 |
| Sarah Kraak | Battista W, Romero-Canyas R, Smith SL, Fraire J, Effron M, Larson-Konar D, et al. Behavior Change Interventions to Reduce Illegal Fishing. Front Mar Sci. 2018;5. doi:10.3389/fmars.2018.00403 |
| Leyre Goti | Bercht AL, Wijermans N. Mind the mind: How to effectively communicate about cognition in social–ecological systems research. Ambio. 2019;48: 590–604. doi:10.1007/s13280-018-1099-7 |
| Sarah Kraak | Bethoney ND, Schondelmeier BP, Kneebone J, Hoffman WS. Bridges to best management: Effects of a voluntary bycatch avoidance program in a mid-water trawl fishery. Marine Policy. 2017;83: 172–178. doi:10.1016/j.marpol.2017.06.003 |
| Sarah Kraak | Bethoney ND, Schondelmeier BP, Stokesbury KDE, Hoffman WS. Developing a fine scale system to address river herring (Alosa pseudoharengus, A. aestivalis) and American shad (A. sapidissima) bycatch in the U.S. Northwest Atlantic mid-water trawl fishery. Fisheries Research. 2013;141: 79–87. doi:10.1016/j.fishres.2012.09.003 |
| Patricia M. Clay | Bisack K, Clay PM. Compliance with marine mammal protection: Focus groups reveal factors in commercial fishermen’s decisions. Marine Policy. 2020;115: 103789. doi:10.1016/j.marpol.2019.103789 |
| Patricia M. Clay | Bisack KD, Das C. Understanding Non-compliance with Protected Species Regulations in the Northeast USA Gillnet Fishery. Front Mar Sci. 2015;2. doi:10.3389/fmars.2015.00091 |
| Kari Stange | Boonstra WJ, Hentati‐Sundberg J. Classifying fishers’ behaviour. An invitation to fishing styles. Fish and Fisheries. 2016;17: 78–100. doi:10.1111/faf.12092 |
| Patricia M. Clay | Bose S, Crees-Morris A. Stakeholder’s views on fisheries compliance: An Australian case study. Marine Policy. 2009;33: 248–253. doi:10.1016/j.marpol.2008.07.004 |
| Sarah Kraak | Drupp MA, Khadjavi M, Quaas MF. Truth-telling and the regulator. Experimental evidence from commercial fishermen. European Economic Review. 2019;120: 103310. doi:10.1016/j.euroecorev.2019.103310 |
| Patricia M. Clay | Gezelius SS. Do Norms Count? State Regulation and Compliance in a Norwegian Fishing Community. Acta Sociologica. 2002;45: 305–314. |
| Patricia M. Clay | Gibson CC, Williams JT, Ostrom E. Local Enforcement and Better Forests. World Development. 2005;33: 273–284. doi:10.1016/j.worlddev.2004.07.013 |
| Patricia M. Clay | Hatcher A, Jaffry S, Thébaud O, Bennett E. Normative and Social Influences Affecting Compliance with Fishery Regulations. Land Economics. 2000;76: 448–461. doi:10.2307/3147040 |
| Sarah Kraak | Holland DS, Martin C. Bycatch Quotas, Risk Pools, and Cooperation in the Pacific Whiting Fishery. Front Mar Sci. 2019;6. doi:10.3389/fmars.2019.00600 |
| Patricia M. Clay | Hønneland G. Compliance in the Barents Sea fisheries. How fishermen account for conformity with rules. Marine Policy. 2000;24: 11–19. doi:10.1016/S0308-597X(98)00058-X |
| Patricia M. Clay | Jentoft S. Legitimacy and disappointment in fisheries management. Marine Policy. 2000;24: 141–148. doi:10.1016/S0308-597X(99)00025-1 |
| Patricia M. Clay | Keane A, Jones JPG, Edwards‐Jones G, Milner‐Gulland EJ. The sleeping policeman: understanding issues of enforcement and compliance in conservation. Animal Conservation. 2008;11: 75–82. doi:https://doi.org/10.1111/j.1469-1795.2008.00170.x |
| Sarah Kraak | Kraak SBM, Bailey N, Cardinale M, Darby C, De Oliveira JAA, Eero M, et al. Lessons for fisheries management from the EU cod recovery plan. Marine Policy. 2013;37: 200–213. doi:10.1016/j.marpol.2012.05.002 |
| Sarah Kraak | Kraak SBM, Hart PJB. Creating a Breeding Ground for Compliance and Honest Reporting Under the Landing Obligation: Insights from Behavioural Science. In: Uhlmann SS, Ulrich C, Kennelly SJ, editors. The European Landing Obligation: Reducing Discards in Complex, Multi-Species and Multi-Jurisdictional Fisheries. Cham: Springer International Publishing; 2019. pp. 219–236. doi:10.1007/978-3-030-03308-8_11 |
| Sarah Kraak | Mackay M, Jennings S, van Putten EI, Sibly H, Yamazaki S. When push comes to shove in recreational fishing compliance, think ‘nudge.’ Marine Policy. 2018;95: 256–266. doi:10.1016/j.marpol.2018.05.026 |
| Ingrid van Putten/Sarah Kraak | Mackay M, van Putten EI, Yamazaki S, Jennings S, Sibly H. Me and My Behavior: An Experiment on Individual Characteristics and Compliance Behavior in Recreational Fishing. Front Mar Sci. 2020;7. doi:10.3389/fmars.2020.579213 |
| Sarah Kraak | Mortensen LO, Ulrich C, Eliasen S, Olesen HJ. Reducing discards without reducing profit: free gear choice in a Danish result-based management trial. ICES Journal of Marine Science. 2017;74: 1469–1479. doi:10.1093/icesjms/fsw209 |
| Patricia M. Clay | Msomphora MR. Stakeholder participation and satisfaction in the process of developing management plans: The case of Scottish Inshore Fisheries Groups. Ocean & Coastal Management. 2015;116: 491–503. doi:10.1016/j.ocecoaman.2015.09.015 |
| Patricia M. Clay | Myers HJ, Moore MJ, Baumgartner MF, Brillant SW, Katona SK, Knowlton AR, et al. Ropeless fishing to prevent large whale entanglements: Ropeless Consortium report. Marine Policy. 2019;107: 103587. doi:10.1016/j.marpol.2019.103587 |
| Patricia M. Clay | Nielsen RJ, Mathiesen C. Important factors influencing rule compliance in fisheries lessons from Denmark. Marine Policy. 2003;27: 409–416. doi:10.1016/S0308-597X(03)00024-1 |
| Sarah Kraak | Needle CL, Dinsdale R, Buch TB, Catarino RMD, Drewery J, Butler N. Scottish science applications of Remote Electronic Monitoring. ICES Journal of Marine Science. 2015;72: 1214–1229. doi:10.1093/icesjms/fsu225 |
| Sarah Kraak | O’Keefe CE, DeCelles GR. Forming a Partnership to Avoid Bycatch. Fisheries. 2013;38: 434–444. doi:10.1080/03632415.2013.838122 |
| Patricia M. Clay | Ostrom E. A General Framework for Analyzing Sustainability of Social-Ecological Systems. Science. 2009;325: 419–422. doi:10.1126/science.1172133 |
| Patricia M. Clay | Pinkerton E, John L. Creating local management legitimacy. Marine Policy. 2008;32: 680–691. doi:10.1016/j.marpol.2007.12.005 |
| Patricia M. Clay | Raakjær Nielsen J. An analytical framework for studying: compliance and legitimacy in fisheries management. Marine Policy. 2003;27: 425–432. doi:10.1016/S0308-597X(03)00022-8 |
| Patricia M. Clay | Röckmann C, Ulrich C, Dreyer M, Bell E, Borodzicz E, Haapasaari P, et al. The added value of participatory modelling in fisheries management – what has been learnt? Marine Policy. 2012;36: 1072–1085. doi:10.1016/j.marpol.2012.02.027 |
| Leyre Goti | Schwarz A-M, Gordon J, Ramofafia C. Nudging statutory law to make space for customary processes and community-based fisheries management in Solomon Islands. Maritime Studies. 2020;19: 475–487. doi:10.1007/s40152-020-00176-0 |
| Sarah Kraak | van Helmond ATM, Chen C, Poos JJ. How effective is electronic monitoring in mixed bottom-trawl fisheries? ICES Journal of Marine Science. 2015;72: 1192–1200. doi:10.1093/icesjms/fsu200 |
| Kari Stange | van Putten I, Longo C, Arton A, Watson M, Anderson CM, Himes-Cornell A, et al. Shifting focus: The impacts of sustainable seafood certification. PLOS ONE. 2020;15: e0233237. doi:10.1371/journal.pone.0233237 |
| Patricia M. Clay | Wiber M, Berkes F, Charles A, Kearney J. Participatory research supporting community-based fishery management. Marine Policy. 2004;28: 459–468. doi:10.1016/j.marpol.2003.10.020 |
| Kari Stange | Wijermans N, Boonstra WJ, Orach K, Hentati‐Sundberg J, Schlüter M. Behavioural diversity in fishing—Towards a next generation of fishery models. Fish Fish. 2020;21: 872–890. doi:10.1111/faf.12466 |
