# Supplementary material for: Behavioural economics in fisheries: A systematic review protocol
Source: PLoS One. 2021 Aug 26;16(8):e0255333. doi: 10.1371/journal.pone.0255333 (PMC8389455; doi:10.1371/journal.pone.0255333)
Supplement: S1 File — (PDF) [file pone.0255333.s006.pdf]

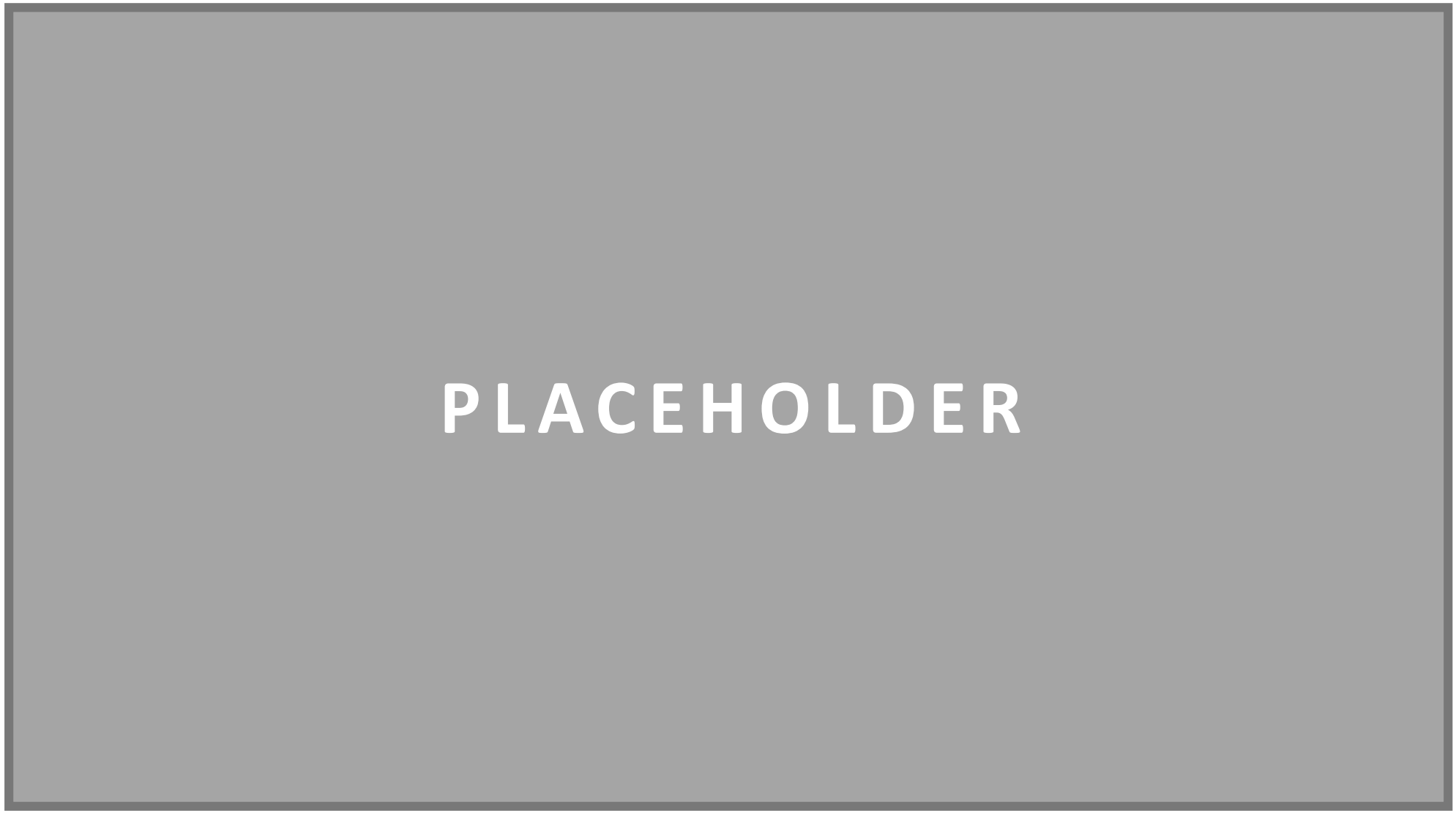


**IMPORTANT: Please note that we are recording this webinar and will distribute it to other stakeholders over the coming days. This also includes the Q&A session and discussion in the end.**

PLACEHOLDER

PLACEHOLDER

# Behavioural Economics in Fisheries

*Systematic Review Protocol*

Alina Wieczorek
Amanda Schadeberg
Julie Krogh Hallin

**Stakeholder Webinar** 25/11/2020 *Microsoft Teams*

Marine Institute
Foras na Mara

WAGENINGEN
UNIVERSITY & RESEARCH

ICES
CIEM
International Council for the Exploration of the Sea
Conseil International pour l'Exploration de la Mer

# Background

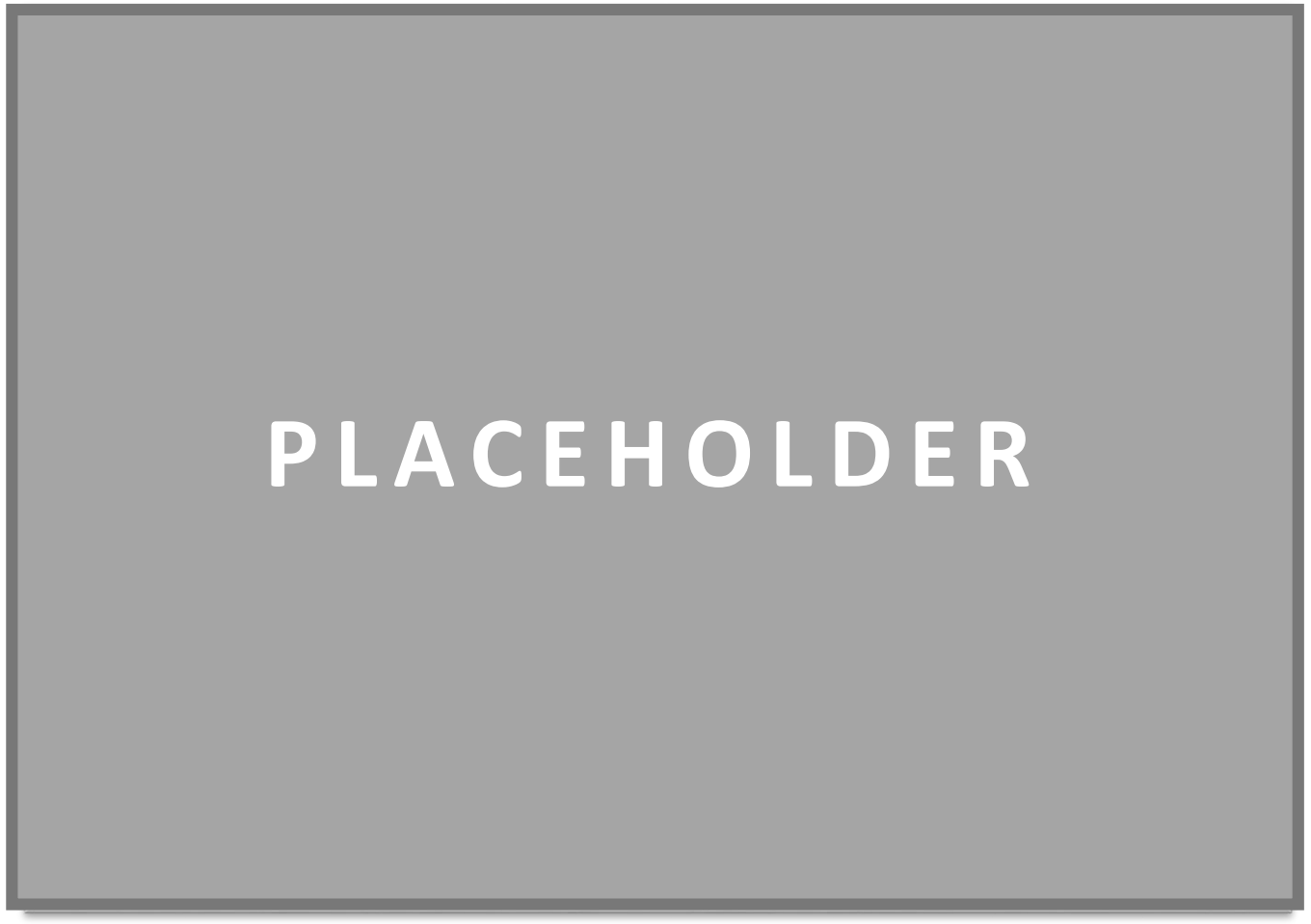


- WGMARS identified BE as an interest area
- BE-group within WGMARS
- Literature review to assess how BE could be used in fisheries
- Call for interested scientist to conduct this study
- We heard you!

# Who we are

## Alina Wieczorek, PhD

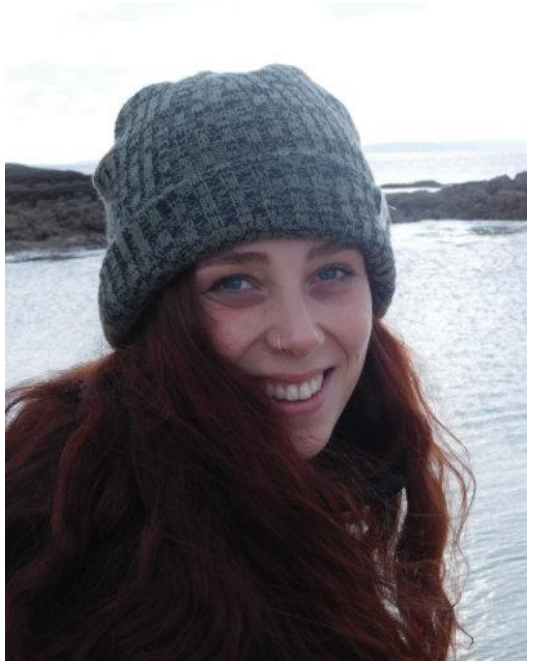

Marine Institute
Foras na Mara

## Amanda Schadeberg

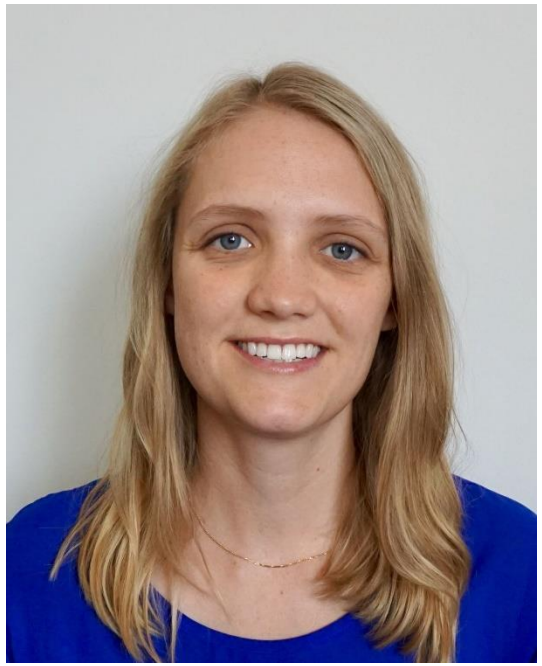

WAGENINGEN
UNIVERSITY & RESEARCH

## Julie Krogh Hallin

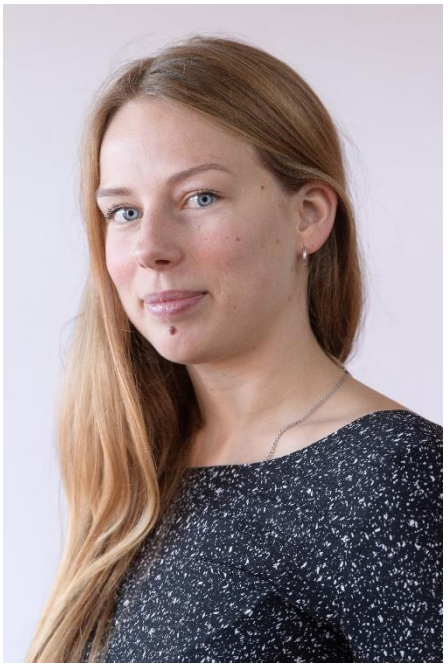

ICES
International Council for the Exploration of the Sea
CIEM
Conseil International pour l'Exploration de la Mer

# Recap on what happened to date

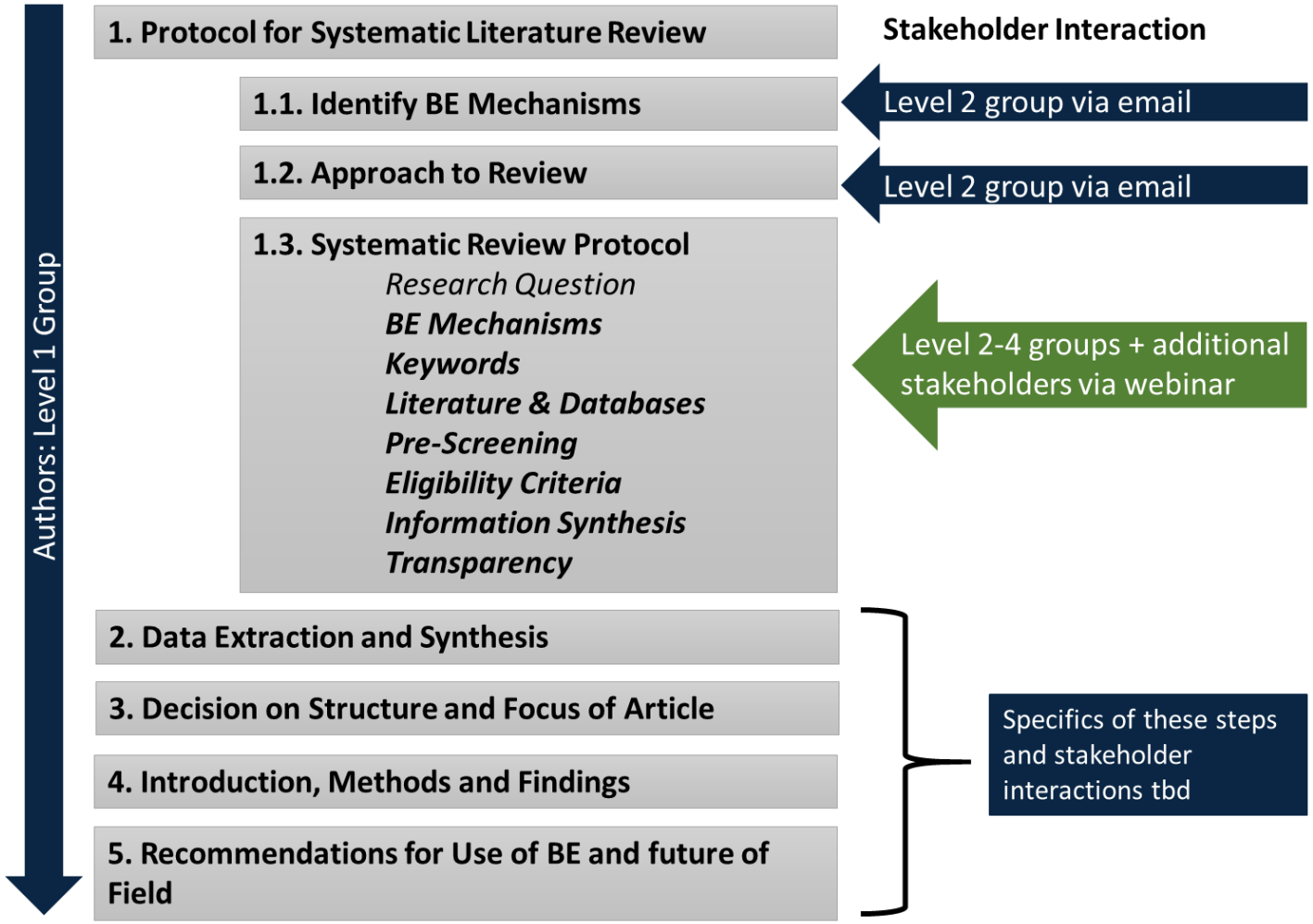


**Level 1**: Alina, Amanda and Julie
**Level 2**: Sarah, Ingrid, Leyre, Andris, Trish and Debbi
**Level 3**: BE interest group
**Level 4**: All WGMARS

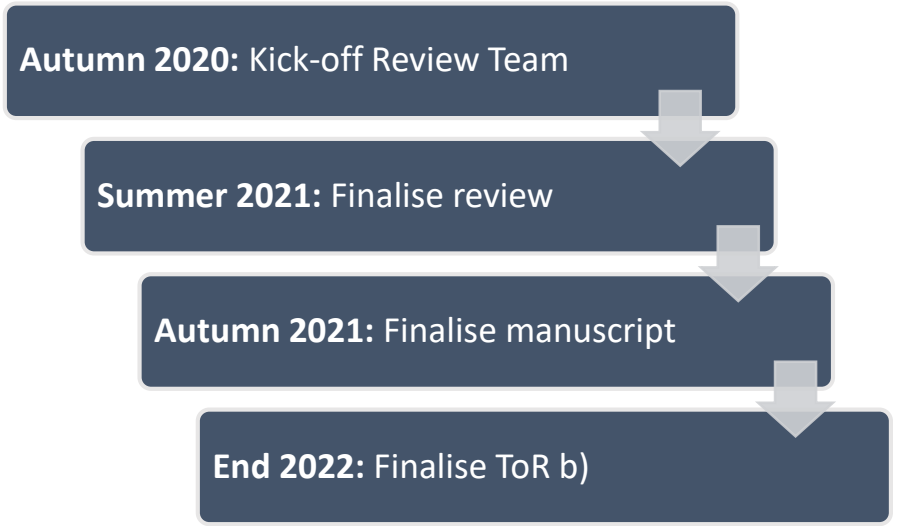

# You as stakeholder

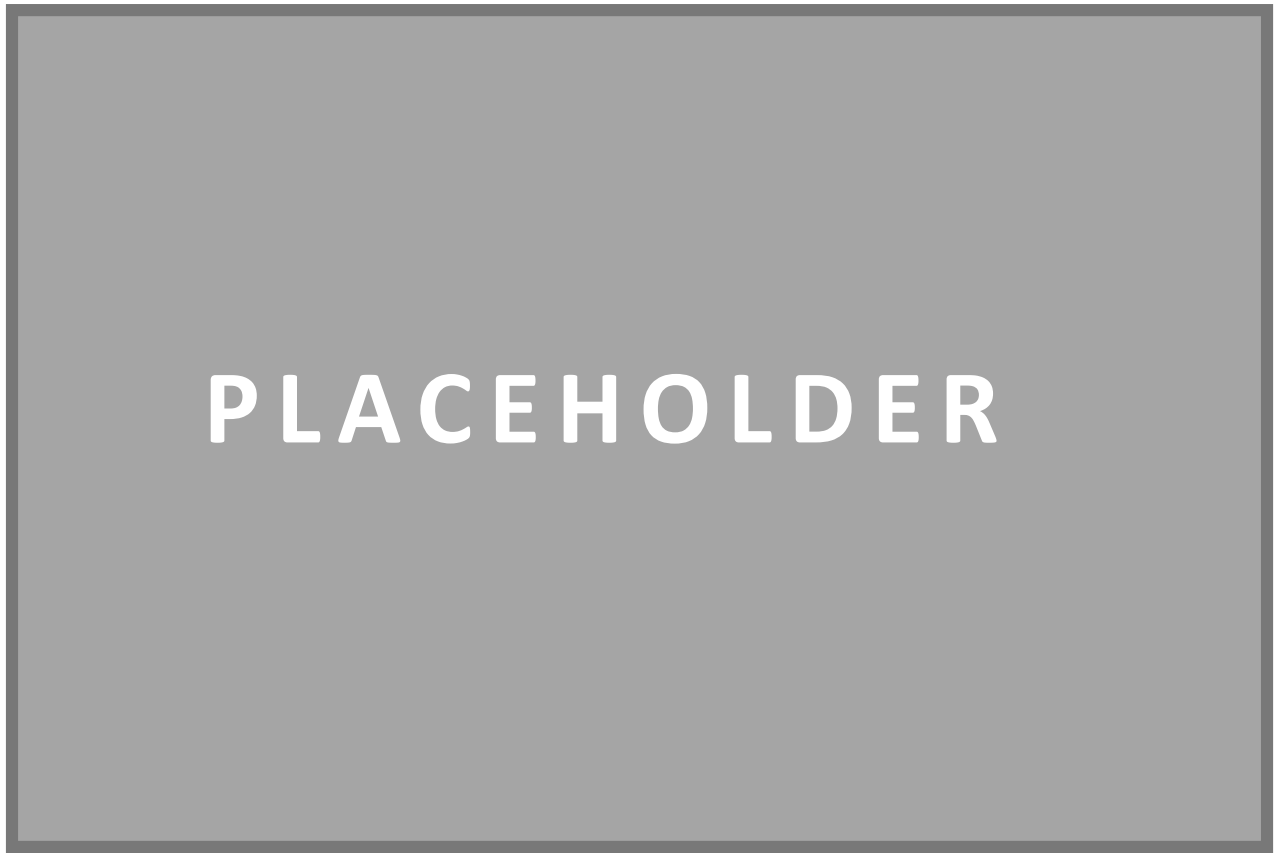


**We want to produce something useful**

- You will help us shape the review study
- Depending on your level your involvement will differ
- The **questionnaire** we will pass on will guide you through the feedback process
- Currently we are in the WGMARS bubble and that may be ok but should also consider other stakeholders – lets discuss in the end

# Research Questions

**Primary Question**

*Option 1:* What principles or mechanisms from behavioural economics are, or could be, useful for the fisheries sector*?

*Option 2:* Which non-financial factors (e.g. social / emotional / cultural) affect fishers' decisions and how could those be leveraged of better management?

**Secondary Questions**

Which mechanisms are applied in the fisheries sector*?

Which mechanisms are effective and how is their effectiveness measured?

At what geographical scale does the study operate?

Which target groups are addressed?

What are ethical implications for the use of behavioural economics in the fisheries sector*?

***fisheries sector** as term to entail all target groups: fishers, traders, retail, consumers, policy makers, law enforcement and scientists

# Mechanism Table

| Mechanism/Driver | Definition/explanation | Example in fisheries | Comment |
|---|---|---|---|
| **Nudge** | Nudging alters people's behavior in a predictable way without forbidding any options or significantly changing their economic incentives. To count as a mere nudge, the intervention must be easy and cheap to avoid. Nudges are not mandates. Putting the fruit at eye level counts as a nudge. Banning junk food does not. (Thaler and Sunstein 2008)[1] | Mackay et al 2018 give several examples of how recreational fishers can be nudged.[2] | Comment: Maybe this should be sub-divided (see 'Rethinking nudge: not one but three concepts' by Mongin and Cozic, 2017) [3] |
| **Loss aversion** | Loss aversion is an important concept associated with prospect theory and is encapsulated in the expression "losses loom larger than gains" (Kahneman & Tversky, 1979). It is thought that the pain of losing is psychologically about twice as powerful as the pleasure of gaining. -www.behavioral economics.com) | A contingent behaviour analysis of 1790 seafood consumers reveals that respondents require a disproportionately large price increase in order to reduce their seafood consumption compared to the price decrease required to induce an equal sized increase in seafood consumption. (Morgan 2008)[4] | It may be hard to separate this from classical economics as it involves a materialistic or financial (direct/indirect) gain. |
| **Reference dependence aka anchoring (see also: shifting baselines)** | The reference (baseline) or status quo sets the point against which changes are seen as 'gains' or losses'. Changing the reference point changes whether people perceive the change as a win or loss. | Working in fishing makes economic agents less risk averse than others. Fishermen also tend to be less sensitive to probability weighting changes in the experiment. | |
| **Non-linear probability weighting** | We overestimate low probability events and underestimate high probability ones (e.g. more afraid of flying than driving, while more likely to die in car accident) | Workers of fisheries sector less risk averse and may have gotten used to risky work environment. (Nguyen & Leung 2009)[5] | |

- Initial literature scanning and level 2 input
- Pre-scoping -> keywords
- 27 mechanisms to date
- **The table is and will remain evolving and organic**
- Are they all relevant?
- Did we miss any?
- Is there overlap?

# Keywords

Fisheries Terms

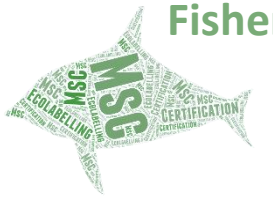


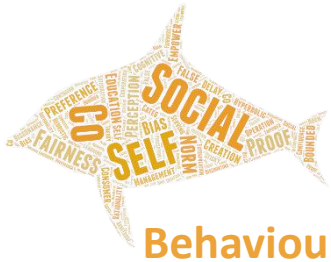


Behavioural Terms

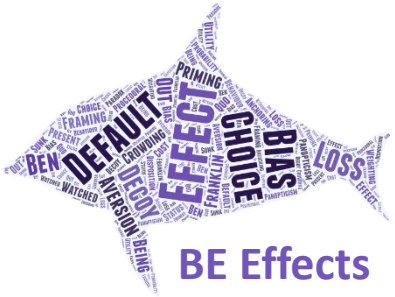


BE Effects

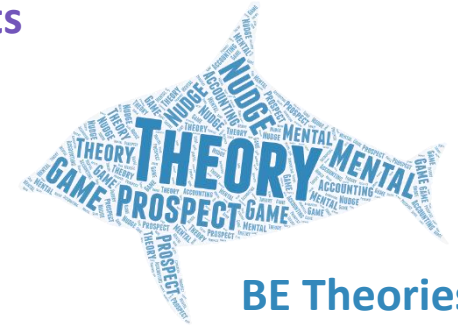


BE Theories

**Keywords to specify for fisheries**

- Fish* is too ambiguous
- Fishing, Seafood, Fisher* work well

**Keywords to specify BE**

- Behavio*
- **AND/OR** Econom*

**Keywords searchstring add-ons**

- Drawn from mechanism table and first literature insights

**Test-Drive**

- 486 hits in Web of Science – Core Collection (select: 'All documents', 'topic=TS')
- First 50 titles very appropriate

**NOTE:** English language only possible – we will acknowledge consequential lack of studies from South America, Asia, Africa etc.

# Databases

PLACEHOLDER

| | |
|---|---|
| **Primary General** | ProQuest |
| | Web of Science – Core Collection |
| | SCOPUS |
| | Science Direct |
| | JSTOR |
| **Fisheries Specific** | Greenfile |
| | Oceanic Abstracts |
| **Social Science** | PsycINFO |
| | SOCA |
| | Social Science Research Network |
| **Economic Specific** | EconBiz |
| | EconLit |
| | EBSCO |
| | RePEc (Research Papers in Economics) |

# Grey Literature

PLACEHOLDER

- Secondary General
  - Digital Dissertation Online (oatd.org)
  - National Academy Press (NAP) – reports
- Specialized Sources for fisheries (from Hughes et al., 2014, Ward-Campbell et al., 2017) and level 1 suggestions
- Literature by Stakeholders (Level 2-4)
- MAYBE: www.google.com; google scholar (need to clear all laptop search history, cookies, caches etc. (info from Haddaway et al., 2015)

**Hughes et al. 2014; Ward-Campbell et al. 2017; Haddaway et al. 2015**

# Screening

PLACEHOLDER

Alina and Julie will independently scan all titles (and abstracts where necessary) for relevance. These lists will be compared and Amanda will act as tie-breaker for the studies where Alina and Julie's assessments do not match

Eligibility Criteria (see next slide)

At this stage add any ‘missed’ mechanisms to table

# Eligibility Criteria

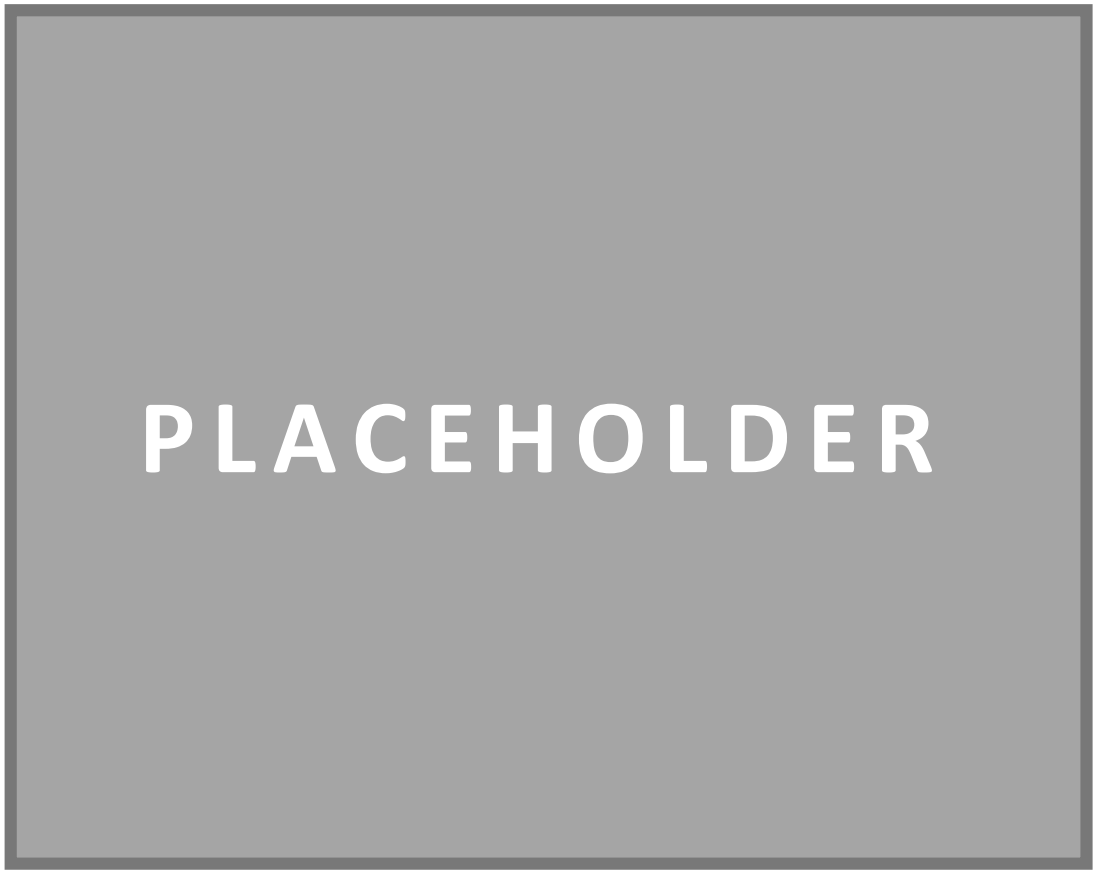


**Eligible populations or subjects**

- Fisheries sector: fishers, traders, retail, consumers, policy makers, law enforcement and scientists
- Any scale and region

**Eligible interventions**

- Interventions that do not directly relate to (indirect) financial gains and losses and thus do not fall in the “classical” economic intervention practice

**Eligible outcomes**

- Any behavioural change in common practice. Any theoretical considerations (e.g. this **could** be done) will be included as well (but recorded as such)

**Eligible type of studies**

- All types of study design will be included with the study type recorded

# Information Synthesis

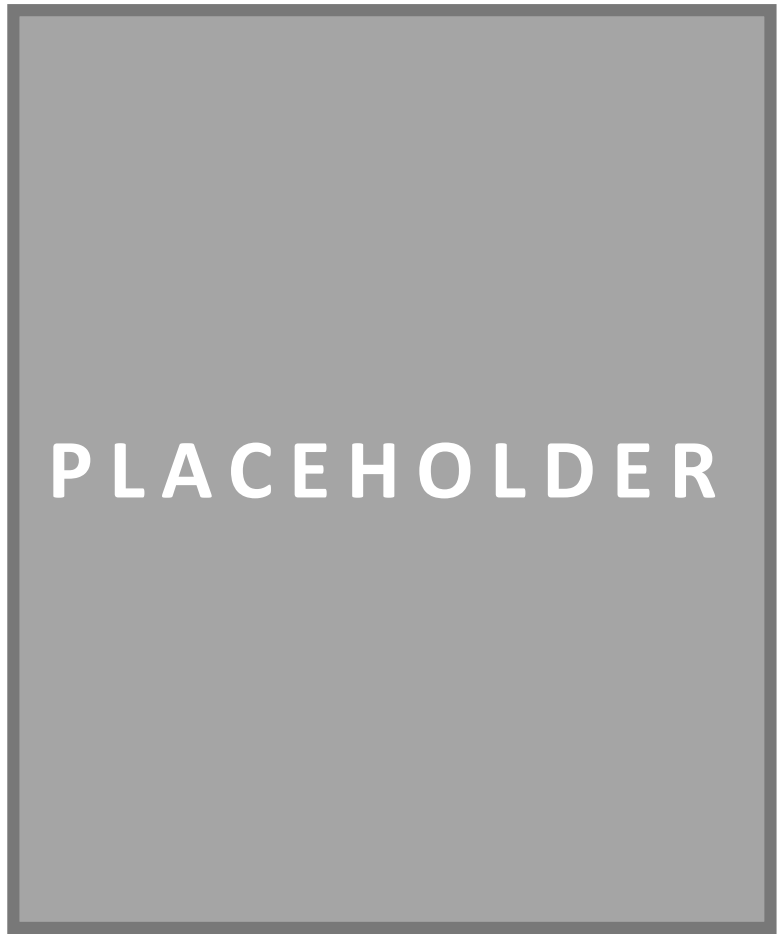


**Literature Type:** Peer-reviewed article, thesis, report, abstracts, proceedings…

**Discipline:** Fisheries, social science, economics…

**Type of study:** Experimental lab/field, review, commentary, foresight…

**Geographic information:** Country, continent, coordinates, GDPR at time of study

**Target group:** Fishers, traders, retail, consumers, policy makers, law enforcement and scientists

**Fishery scale:** Artisanal (e.g. vessel <10m), small scale (10m-20m), large scale (20m-30m), industrial scale (30m+)

**Study focus:** E.g. gear, safety, TAC/quota, area, seafood/consumer

**Behavioural mechanism:** From mechanism table

**Outcome:** Result and considerations for future

**Ethical consideration:** Were ethics considered and if so, what was the view on this?

# Registration and Protocol Publication

PLACEHOLDER

1. Register Protocol* using ROSES *pro forma* (Haddaway et al. 2018) and register it with www.osf.io (alternative is PROSPERO but seems to be purely medical plus COVID delays)
2. Submit protocol to:
   - Systematic reviews (would bind us to publish in this journal for later publication but seems very suitable)
   - Nature protocols –has no SR as of now
   - PLOS One has published SR protocols as well
   - Alternatives?

***Allers et al. 2017 – arguments for registration of SR**

# What do we need from you?

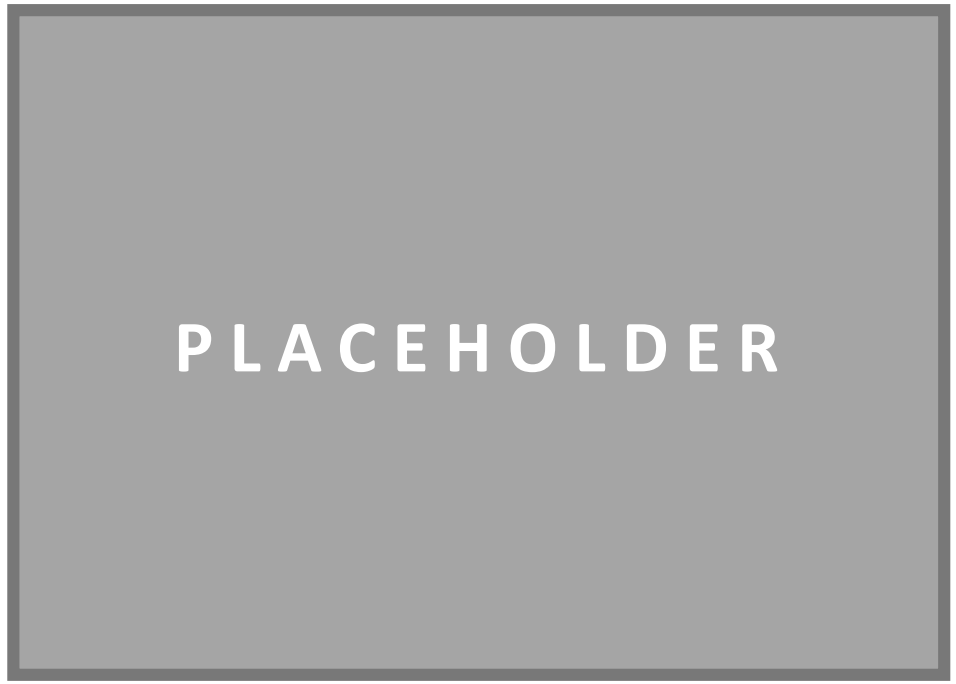


**What:** Provide some written feedback on our progress so far via the questionnaire (link below, and in the chat)

**Who**: All of you attending, and any other relevant stakeholders we will identify

**When**: Preferably **before Friday 4th of December**, so we can begin to finalise the protocol (if later please email to let us know)

**Questionnaire:**
https://mws.onlinesurveys.ac.uk/behavioural-economics-in-fisheries-systematic-review-proto-2

# THANK YOU!

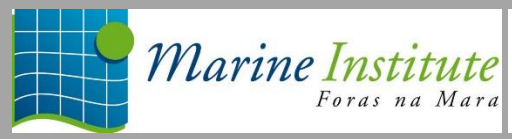
Marine Institute
Foras na Mara

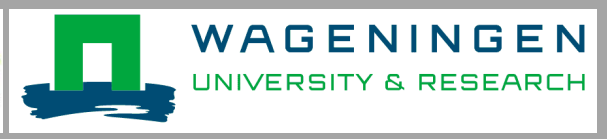
WAGENINGEN
UNIVERSITY & RESEARCH

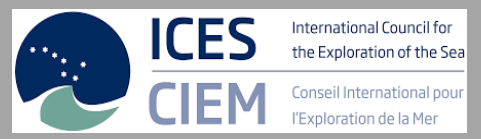
ICES
International Council for the Exploration of the Sea
CIEM
Conseil International pour l'Exploration de la Mer
