# Supplementary material for: Behavioural economics in fisheries: A systematic review protocol
Source: PLoS One. 2021 Aug 26;16(8):e0255333. doi: 10.1371/journal.pone.0255333 (PMC8389455; doi:10.1371/journal.pone.0255333)
Supplement: S2 File — (PDF) [file pone.0255333.s007.pdf]

Behavioural Economics in Fisheries Systematic Review Protocol Feedback (copy)

Page 1: Introduction

Welcome,

This questionnaire was designed as a follow-up from a webinar which was given live on the 25th of November 2020.

The webinar introduced the concept of a systematic review on the use of behavioural economics in fisheries which will be conducted in the context of the behavioural economics task group of the ICES WGMARS working group.

If you were not able to attend you can find a recording of the webinar in the ICES WGMARS SharePoint in the 'BE-group work in progress' folder.

If you do not have access to this folder or have difficulties locating the webinar in the SharePoint please contact Alina Wieczorek (Alina.Wieczorek@marine.ie) who will be able to pass you on a download link.

Once you have watched the webinar we would kindly invite you to take part in the questionnaire as a stakeholder for the systematic review.

In the questionnaire we will be asking you for your opinion on the scope and specific aspects of the review. This will allow us to produce a product which will be most useful for you as a stakeholder and future user of the review.

We may want to follow-up on some of your suggestions in the future. For that reason, we will be collecting some of your personal details. Please note the disclaimer below before proceeding with the survey.

Thank you very much for your interest in this work and we are looking forward to receiving your feedback.

Alina Wieczorek
Amanda Schadeberg
Julie Krogh Hallin

By proceeding with the questionnaire you are giving your consent and you agree to voluntarily provide your personal data. You have the right to revoke the given consent. The personal information provided by you will be stored on a password-protected file and used solely for the purpose for which you have provided these. You have the right to inspect, delete, correct, and restrict the processing of personal data as well as the right to appeal and the right to data portability https://www.marine.ie/Home/site-area/online-policies/privacy. For further information you can contact the Marine Institute via dpo@marine.ie.

Page 2: Personal Information

1. **First Name** ✱ *Required*

2. **Surname** ✱ *Required*

3. **Affiliation** ✱ *Required*

4. Country ✱ *Required*

5. E-Mail Address ✱ *Required*

6. Stakeholder Group ✱ *Required*

- [ ] Social Scientist
- [ ] Natural Scientist
- [ ] Economist
- [ ] Decision Maker
- [ ] Other

6.a. If you selected Other, please specify:

Page 3: Research Question

In the following we have listed proposed primary and secondary research questions for the systematic review. These guide the scoping of the review. While it was decided that there will be a focus on mechanisms used in behavioural economics these questions are of now a draft and can change with the input of potential future users (you). We would therefore like to invite you to give feedback on whether you think addressing these questions could lead to answers which will be of use for the fisheries management sector in the future.

Primary Research Question

Option 1: What principles or mechanisms from behavioural economics are, or could be, useful in the fisheries sector*?

Option 2: Which non-financial factors (e.g. social / emotional / cultural) affect fishers' decisions and how could those be leveraged for better management?

Secondary Questions

Which mechanisms are applied in the fisheries sector*?

Which mechanisms are effective and how is their effectiveness measured?

At what geographical scale does the study operate?

Which target groups are addressed?

What are ethical implications for the use of behavioural economics in the fisheries sector*?

*fisheries sector is used as a term to entail all target groups: fishers, traders, retail consumers, policy makers, law enforcement and scientists

7. Which primary research question (option 1 or 2) would you prefer, and why? Could you maybe think of a better one? ✱ *Required*

8. Do you have any suggestions for additional secondary questions, or would you formulate any of the secondary questions differently? *Optional*

Page 4: Behavioural Economics Mechanisms

We have begun working on a summary of different mechanisms and examples from (behavioural) economics in order to guide our systematic review. It is by no means complete. We would like to ask you to browse the table and, if possible, let us know whether there are any obvious omissions. Are there concepts, mechanisms, or key examples from the literature that we appear to have missed?

Mechanism/Driver	Definition/explanation	Example in fisheries	Comment
**Nudge**	Nudging alters people's behavior in a predictable way without forbidding any options or significantly changing their economic incentives. To count as a mere nudge, the intervention must be easy and cheap to avoid. Nudges are not mandates. Putting the fruit at eye level counts as a nudge. Banning junk food does not. (Thaler and Sunstein 2008)[1]	Mackay et al 2018 give several examples of how recreational fishers can be nudged.[2]	Comment: Maybe this should be sub-divided (see 'Rethinking nudge: not one but three concepts' by Mongin and Cozic, 2017) [3]
**Loss aversion**	Loss aversion is an important concept associated with prospect theory and is encapsulated in the expression "losses loom larger than gains" (Kahneman & Tversky, 1979). It is thought that the pain of losing is psychologically about twice as powerful as the pleasure of gaining.<br>-www.behavioral economics.com)	A contingent behaviour analysis of 1790 seafood consumers reveals that respondents require a disproportionately large price increase in order to reduce their seafood consumption compared to the price decrease required to induce an equal sized increase in seafood consumption. (Morgan 2008)[4]	It may be hard to separate this from classical economics as it involves a materialistic or financial (direct/indirect) gain.
**Reference dependence aka anchoring (see also: shifting baselines)**	The reference (baseline) or status quo sets the point against which changes are seen as 'gains' or losses'. Changing the reference point changes whether people perceive the change as a win or loss.	Working in fishing makes economic agents less risk averse than others. Fishermen also tend to be less sensitive to probability weighting changes in the experiment.	

**Non-linear probability weighting**	We overestimate low probability events and underestimate high probability ones (e.g. more afraid of flying than driving, while more likely to die in car accident)	Workers of fisheries sector less risk averse and may have gotten used to risky work environment. (Nguyen & Leung 2009)[5]
**Decoy effect**	Offering a bad choice in the list of options prompts people to accept paying a higher price than they might have without the comparable options	None found in informal literature screening but fish market pricing may be a good example.
**Using defaults (eg. Opt in/out)**	Making the preferred option the default increases the number of people who choose it, because opting out requires conscious effort	None found in informal literature screening.
**Social Norms**	Social norms signal appropriate behavior and are classed as behavioral expectations or rules within a group of people (Dolan et al., 2010).<br><br>-www.behaviouraleconomics.com	Mackay et al. (2019) use descriptive social norm to nudge recreational fishers to stick to bag limit. [6]
**Social proof**	The influence exerted by others on our behavior can be expressed as being either normative or informational. Normative influence implies conformity in order to be accepted or liked (Aronson et al., 2005), while informational influence occurs in ambiguous situations where we are uncertain about how to behave and look to others for information or cues. www.behaviouraleconomics.com	Observational example from Ireland: Fishers priding themselves to dispose of lost gear accordingly and to commit to retrieving lost gear.<br><br>Also fishers providing information and participating in scientific studies.
**Self-image**	From van der Werff & Keizer 2013: Environmental self-identity is related to one's obligation-based intrinsic motivation (that is, feelings of moral obligation) to act pro-environmentally, which in turn affects pro-environmental actions. [7]	Modifying social norms can create conditions that incentivize a company, country, or individual to fish sustainably, curb illegal fishing, or create large marine reserves as steps to enhance reputation or self-image.

**Priming**	Behavioral priming refers to the notion that exposing people to an external stimulus (e.g., a list of words describing old people) activates a mental construct associated with this stimulus (e.g., “being old”), which may in turn affect overt behavior without the actor necessarily being aware of this influence	From Kraak & Hart (2019) referring to Drupp et al. (2019): Fishers misreported coin tosses to their economic advantage more strongly in a treatment where they were faced with the EU logo. Fishers were more honest in an additional treatment where the source of research funding, namely the EU, was revealed. These apparently contradictory findings suggest that lying is increased towards a disliked regulator, but perhaps decreased when it is made clear from whom the money is “stolen”. [8,9]
**Framing**	The framing effect is a cognitive bias where people decide on options based on whether the options are presented with positive or negative connotations; e.g. as a loss or as a gain. People tend to avoid risk when a positive frame is presented but seek risks when a negative frame is presented	Framing economic incentives for fishers differently has different results as shown in study on small-scale Turkish fisheries. (Ertör-Akyazi 2019)[10]
**Knowledge-based decision-making**<br>**OR**<br>**Knowledge effect on decision-making**	Education/knowledge/awareness has a large impact on decision-making	Educated seafood consumer choices (e.g.: Jodice et al. 2020)[11]
**Involving People in problem solving/ Empower People/Co-creation**	Developing solutions to specific problems/actions WITH the target group.	Understanding fishers gear choice and communicating environmental concerns to them in order to co-manage schemes effective in tropical small-scale fisheries (Herron et al. 2020) [12]

**Effect of being watched/panopticism**	When you know you are being watched, but not when or by whom, your compliant behaviour rises overall (Foucault/Bentham theory of the panopticon). i.e. the possibility of being watched makes people behave *as if* they are being watched (whether they are watched or not)	AIS, Drones etc. (Kraak & Hart 2019)[8]	
**Hyperbolic discounting**	Hyperbolic discounting refers to the tendency for people to increasingly choose a smaller-sooner reward over a larger-later reward as the delay occurs sooner rather than later in time.<br><br>-www.behaviorlab.org	Managing harvesting times in fisheries (e.g. Duncan et al. 2011)[13]	
**Hedonic Framing**	Hedonic framing refers to how people try to maximise psychological pleasure and minimise pain (regret) when faced with decisions relating to gains and losses. This means that two individual gains are perceived to be more valuable than a single larger gain of the same value.<br><br>-www.conversion-uplift.co.uk	None found in informal literature screening but an example could be area closure/opening (i.e. opening it twice a season rather than for a longer period at a time).	
**Endowment Effect**	The endowment effect refers to an emotional bias that causes individuals to value an owned object higher, often irrationally, than its market value.<br><br>-www.dobodot.com	None found in informal literature screening but examples could include ‘owning’ specific areas for fishing and that fishers would prefer to stick to those even if newly allocated ones may be more productive/yield higher catch.	Related to loss aversion

**Disposition Effect**	The disposition effect refers to investors' reluctance to sell assets that have lost value and greater likelihood of selling assets that have made gains (Shefrin & Statman, 1985). This phenomenon can be explained by prospect theory (loss aversion), regret avoidance and mental accounting.<br><br>-www.behavioraleconomics.com		
**Crowding Out of Compliance**	Control imposed by an outside institution undermines – "crowds out" – any intrinsic motivations an individual may have to comply voluntarily. (Kraak & Hart 2019)[8]	Kraak & Hart (2019) referring to Ostrom (2009): 'Self-imposed rules, self-imposed monitoring and self-imposed sanctions work better.[14]	
**Paradox Choice**	The paradox of choice, popularized by psychologist Barry Schwartz in a 2004 book, is the theory that having more options, or choices, makes it harder for people to make a decision, potentially hurting their well-being in the process. The theory has been tested and analyzed in many different ways over the years.<br><br>-www.zachhellermarketing.com	None found in informal literature screening but an example could be seafood choice in supermarkets/fish mongers – we now have global markets and there may be 5 different types of Salmon which makes it harder to make the right decision (whether this is how they are farmed, where they are farmed etc.)	
**Present Bias**	Present bias is the tendency to rather settle for a smaller present reward than to wait for a larger future reward, in a trade-off situation. It describes the trend of overvaluing immediate rewards, while putting less worth in long-term consequences.<br><br>-Wikipedia	None found in informal literature screening but an example could be the timing when fishers return to port. They could go out to sea and catch a decent (but not huge) amount and may decide to return to port even if conditions look promising in the following days (in which case initial catch may be illegally dumbed?).	Present bias = Hyperbolic discounting?

**Procedure Variability**	People not only care about outcomes; they also value the procedures that lead to the outcomes. Procedural utility is an important source of human well-being. (Frey et al. 2004)[15]	None found in informal literature screening but an example could be the process of closing an area for fisheries or the way new gear is implemented.	
**Status-quo bias**	Status quo bias is evident when people prefer things to stay the same by doing nothing (see also inertia) or by sticking with a decision made previously (Samuelson, & Zeckhauser, 1988). This may happen even when only small transition costs are involved and the importance of the decision is great.<br><br>-www.behavioraleconomics.com	Should we include this? I think it may be relevant but could not think of an example.	Related to loss aversion
**Sunk cost fallacy**	Individuals commit the sunk cost fallacy when they continue a behavior or endeavor as a result of previously invested resources (time, money or effort) (Arkes & Blumer, 1985). This fallacy, which is related to loss aversion and status quo bias, can also be viewed as bias resulting from an ongoing commitment.<br><br>-www.behavioraleconomics.com	None found in informal literature screening but an example could be to stick to more harmful fishing gear even if newer gear is shown to give higher yield and be more environmentally friendly.	Related to loss aversion
**Incentive**	(Financial) Incentives which will be followed according to system 2.	Subsidies<br><br>Bonds/insurance<br><br>Quota<br><br>Revenue from renting out quota<br><br>Trade<br><br>-> Numerous examples in literature	This ‘classical’ economic approach does not necessarily fall in the scope of this review but is included here to get a broad overview

**Punishment**	(Financial) Punishment which will be avoided according to system 2.	Fines<br><br>Trade tariffs<br><br>Tax/levies<br><br>Spatial restrictions/closures<br><br>Loss of license<br><br>-> Numerous examples in literature	This ‘classical’ economic approach does not necessarily fall in the scope of this review but is included here to get a broad overview

9. **Please type any suggestions in the box below. If referring to literature, please give a complete title or a digital object identifier (DOI).** ✱ *Required*

Page 5: Keywords

We have chosen some potential keywords. The process of identifying keywords was guided by the research questions and the mechanism table. These are by no means final. In order to identify the correct keywords and to ensure a complete and unbiased literature search, we ask you to browse the list and let us know what you think of the current selection and if you noticed any obvious omissions.

Initial Brainstorming	Logical Analysis	First Assessment	Current Keyword Pool
Anchoring	Good	Inclusion	Anchoring
Arbitrage	Good	Inclusion	Arbitrage
Asymmetric information	Too vague	Exclusion	
Being watched	Good	Inclusion	Being watched
Ben Franklin effect	Good	Inclusion	Ben Franklin effect
Bonds/insurance	Classical Economics	Classical Economics	
Bounded rationality	Good	Inclusion	Bounded rationality
Co-Creation	Good	Inclusion	Co-Creation
Cognitive bias	Good	Inclusion	Cognitive bias
Co-Management	Good	Inclusion	Co-Management
Competition	Too ambiguous	Exclusion	
Complian	Too broad	Exclusion	
Consumer confidence	Good	Inclusion	Consumer confidence
Co-operation/cooperation	Good	Inclusion	Co-operation/cooperation
Crowding out	Good	Inclusion	Crowding out
Decision	Too broad	Exclusion	
Decoy effect	Good	Inclusion	Decoy effect
Default behaviour	Good	Inclusion	Default behaviour
Default choice	Good	Inclusion	Default choice
Disposition effect	Good	Inclusion	Disposition effect
Dissonance	Good	Inclusion	Dissonance
Ecolabelling	Good	Inclusion	Ecolabelling
Education	Good	Inclusion	Education
Empower	Good	Inclusion	Empower
Equity	Unsure if it falls in scope	Maybe	
Fairness/Perception of fairness	Good	Inclusion	Fairness/Perception of fairness
False extrapolation	Good	Inclusion	False extrapolation
Fines	Classical Economics	Classical Economics	
Framing	Good	Inclusion	Framing
Game theory	Good	Inclusion	Game theory
Heuristic	Good	Inclusion	Heuristic
Hyperbolic discounting	Good	Inclusion	Hyperbolic discounting

Incentive	Classical Economics	Classical Economics	
Inertia	Probably too general	Exclusion	
Insight	Too broad	Exclusion	
Intuition	Probably too general	Exclusion	
Knowledge	Too ambiguous, more specific terms will be better	Exclusion	
Loss aversion	Good	Inclusion	Loss aversion
Loss of license….	Classical Economics	Classical Economics	
Melioration theory	Classical Economics	Classical Economics	
Mental accounting	Good	Inclusion	Mental accounting
MSC certification	Good	Inclusion	MSC certification
Nudge	Good	Inclusion	Nudge
Opt in/out	Good, but may need rewording	Maybe	
Panopticism	Good	Inclusion	Panopticism
Paradox choice	Good	Inclusion	Paradox choice
Preference	Good	Inclusion	Preference
Premium	Classical Economics	Classical Economics	
Present bias	Good	Inclusion	Present bias
Priming	Good	Inclusion	Priming
Probability weighting	Good	Inclusion	Probability weighting
Procedural utility	Good	Inclusion	Procedural utility
Prospect theory	Good	Inclusion	Prospect theory
Punish	Classical Economics	Classical Economics	
Quota	Classical Economics	Classical Economics	
Revenue from renting out quota	Classical Economics	Classical Economics	
Satisfaction delay	Good	Inclusion	Satisfaction delay
Self perception	Good	Inclusion	Self perception
Self serving	Good	Inclusion	Self serving
Self theory	Good	Inclusion	Self theory
Shift	Probably too general	Exclusion	
Social category	Good	Inclusion	Social category
Social contagion	Good	Inclusion	Social contagion
Social learning	Good	Inclusion	Social learning
Social Norm	Good	Inclusion	Social Norm
Social Proof	Good	Inclusion	Social Proof

Spatial restrictions/closures	Classical Economics	Classical Economics	
Status-quo bias	Good	Inclusion	Status-quo bias
Sticky preference	Good	Inclusion	Sticky preferance
Subsidy	Classical Economics	Classical Economics	
Sunk cost	Good	Inclusion	Sunk cost
Tax/levies	Classical Economics	Classical Economics	
Trade	Classical Economics	Classical Economics	
Trade tariffs	Classical Economics	Classical Economics	
Using defaults	Good, but better if more specificwording (see below)	Exclusion	

AND fisher* OR fishing OR seafood
AND econom*
AND behavio*

10. Do you think the keywords we have listed here will result in literature findings addressing our research questions? ✱ *Required*

- ◯ Yes
- ◯ No

11. If you selected "No" in the previous question, please explain your answer. *Optional*

12. Is there an important keyword relating to our research question that we appear to have missed?

Keyword 1

13. **Keyword 2**

14. **Keyword 3**

15. **Keyword 4**

16. **Keyword 5**

17. **Keyword 6**

18. **Keyword 7**

19. **Keyword 8**

Page 6: Literature

Databases

Below you will find a list of databases which is a pre-selection we made based on those we have found to be re-current in systematic reviews which investigate fisheries, social science and economic topics.

As noted by Bramer et al. (2017) including too many databases will make the review a laborious task and brings some complications with it. We therefore would like to include only as many databases as it is necessary.

Please have a look at the selection and select the databases you think we should focus on. Please use the "Other" option to let us know whether there are any obvious omissions.

20. **Primary General** ✱ *Required*

- ☐ ProQuest
- ☐ Web of Science – Core Collection
- ☐ SCOPUS
- ☐ Science Direct
- ☐ JSTOR
- ☐ Other

20.a. If you selected Other, please specify:

21. **Fisheries/Environmental Specific** ✱ *Required*

- ☐ Greenfile
- ☐ Oceanic Abstracts
- ☐ I don't know
- ☐ Other

21.a. If you selected Other, please specify:

22. **Social Science Specific** ✱ *Required*

- ☐ PsycINFO
- ☐ SOCA
- ☐ Social Science Research Network
- ☐ I don't know
- ☐ Other

22.a. If you selected Other, please specify:

23. **Economic Specific** ✱ *Required*

- ☐ EconBiz
- ☐ EconLit
- ☐ EBSCO
- ☐ RePEc (Research Papers in Economics)
- ☐ I don't know
- ☐ Other

23.a. If you selected Other, please specify:

Grey Literature

We have decided to include grey literature (reports, dissertations, anecdotal observations) in the review.

We will be screening the following two databases for these:

- Digital Dissertation Online (oatd.org)
- National Academy Press (NAP) – reports

Furtermore we will also hand-search the following webpages which have been either used within previous related literature reviews or have been nominated within our team:

- Centre for Environment, Fisheries and Aquaculture
- Commonwealth Scientific and Insustrial Research (Australia)
- Department of the Environment, Food and Rural Affairs (UK)
- Environment Agency
- European Environment Agency
- Fisheries and Oceans Canada
- Food and Agriculture Organization of the United Nations
- Joint Nature Conservation Committee
- National Institute of Water and Atmospheric Research
- Nature Conservacy
- United Nations Environment Programme
- US Fish and Wildlife Service
- ICES publications/library
- Alaska Seafood Cooperative
- Centre for Environment, Fisheries and Aquaculture Science
- Commonwealth Scientific and Industrial Research Organisation
- Department of Fisheries and Oceans Canada
- Food and Agriculture Organization of the United Nations
- Fisheries Research Service Scotland
- Marine Institute Ireland
- French Research Institute for Exploitation of the Sea
- Marine Stewardship Council
- National Oceanic Atmospheric Administration
- Northern Ireland Environment Agency
- National Institute of Water and Atmospheric
- North Pacific Marine Science Organization
- Northwest Atlantic Fisheries Organization
- World Wide Fund for Nature
- Australian Government Guide to Behavioural Economics (toolkit)
- Behavioraleconomics.com
- Behaviouralinsights.nl

24. **We feel that we may be missing some non-European fisheries related sources as well as economic sources. Please scan the above list and let us know whether there are any obvious omissions.** *
Required

Stakeholder Literature Suggestions

As you are a stakeholder and potential future user of this study, we would like to invite you to submit specific literature suggestions.

25. **If you have literature to suggest, please provide us with either the full title, a doi code or a relevant web link.** ✱ *Required*

Page 7: Screening, Eligibility Criteria and Information Synthesis

Screening

For the screening process we will follow the steps outlined in the ROSES *pro forma* diagram as presented during the webinar.

ROSES Flow Diagram for Systematic Reviews. Version 1.0

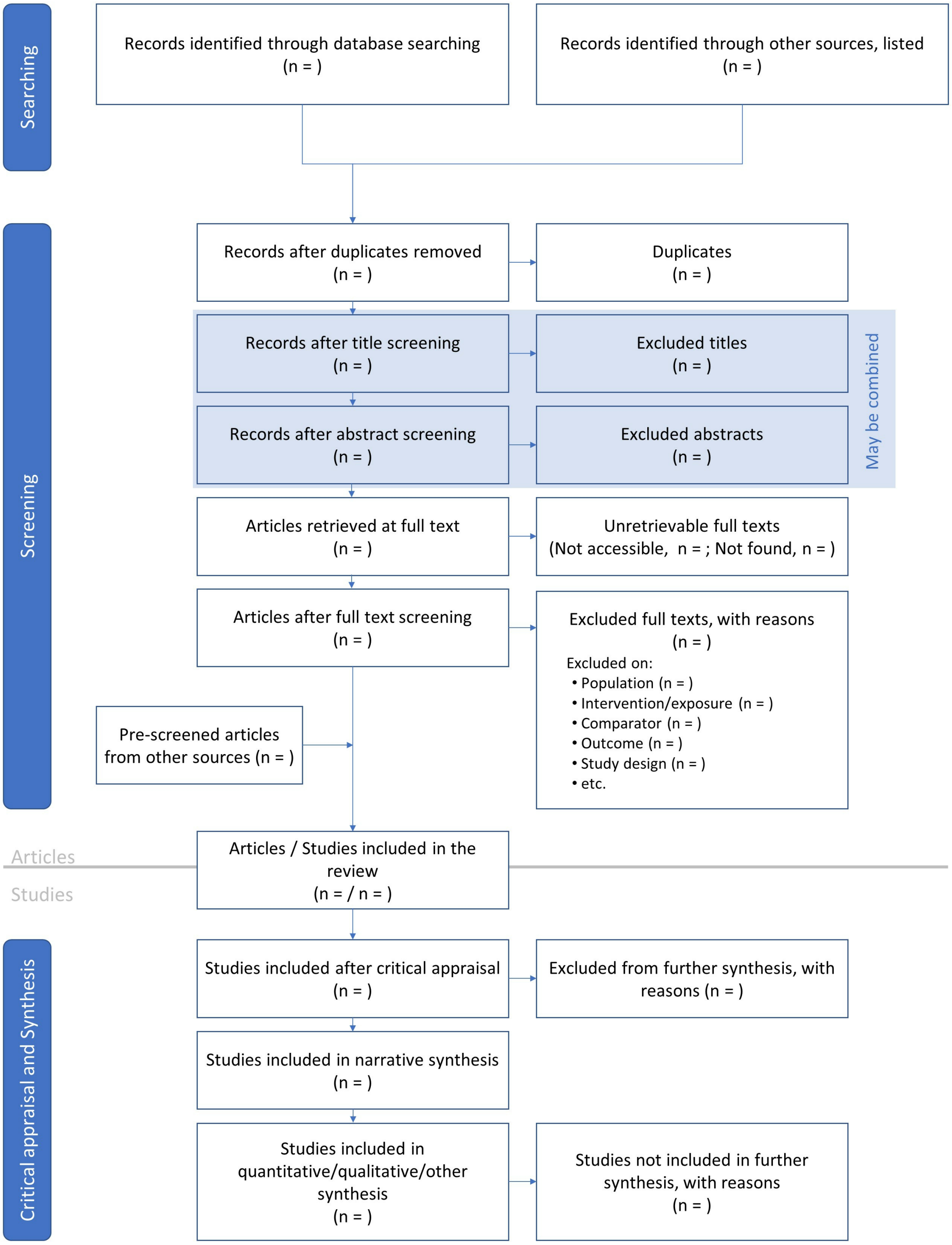

26. **If you have any feedback on this process please leave a comment here.** *Optional*

Eligibility Criteria

Please have a look at the eligibility criteria we have devised so far.

Eligible populations or subjects

- Fisheries sector: fishers, traders, retail, consumers, policy makers, law enforcement and scientists
- Any scale and region

Eligible interventions

- Interventions that do not directly relate to (indirect) financial gains and losses and thus do not fall in the “classical” economic intervention practice
- We will use keywords to focus on the mechanisms identified in the table, but we will also consider mechanisms that emerge from the review

Eligible outcomes

- Any behavioural change in common practice
- Any theoretical considerations (e.g. this study/intervention could be done) will be included (but recorded as such)

Eligible type of studies

- All types of study design will be included with the study type recorded.

27. **These eligibility criteria will narrow the scope of the study but also ensure an unbiased literature review and are therefore very important.**

Please let us know if you have any comments or suggestions we should consider in these criteria.
Optional

Information Synthesis

We plan to synthesise the following information from the screened articles:

Literature Type: Peer-reviewed article, thesis, report, abstracts, proceedings…

Discipline: Fisheries, social science, economics…

Type of study: Experimental lab/field, review, commentary, foresight…

Geographic information: Country, continent, coordinates, GDPR at time of study

Target group: Fishers, traders, retail, consumers, policy makers, law enforcement and scientists

Fishery scale: Artisanal (e.g. vessel <10m), small scale (10m-20m), large scale (20m-30m), industrial scale (30m+)

Study focus: E.g. gear, safety, TAC/quota, area, seafood/consumer, etc.

Behavioural mechanism: From mechanism table

Outcome: Result and considerations for future

Ethical consideration: Were ethics considered and if so what was the view on this?

28. **Please leave any comments on this structure of extracting information from the articles in the box below.** *Optional*

Page 8: Anything we have missed?

29. **Is there anything else you would like to add about the work so far that hasn't been addressed in the questionnaire?** *Optional*

Page 9: Thank you very much for completing the questionnaire.

Your input and insights as stakeholder of this systematic review are very important to ensure that the study and its outcomes will be of use to you and the wider community.

We thank you for taking the time to go through this questionnaire and hope this will allow us to finalise our systematic review protocol in due course.

Best Wishes,

Alina, Amanda and Julie

Literature referred to in the questionnaire

(1) “Nudge: improving decisions about health, wealth, and happiness” by Richard H. Thaler and Cass R. Sunstein https://www.consilium.europa.eu/en/documents-publications/library/library-blog/posts/nudge-improving-decisions-about-health-wealth-and-happiness/ (accessed Nov 23, 2020).

(2) Mackay, M.; Jennings, S.; van Putten, E. I.; Sibly, H.; Yamazaki, S. When Push Comes to Shove in Recreational Fishing Compliance, Think ‘Nudge.’ *Marine Policy* **2018**, *95*, 256–266. https://doi.org/10.1016/j.marpol.2018.05.026.

(3) Mongin, P.; Cozic, M. Rethinking Nudge: Not One but Three Concepts. *Behavioural Public Policy* **2018**, *2* (1), 107–124. https://doi.org/10.1017/bpp.2016.16.

(4) Morgan, A. Loss Aversion and a Kinked Demand Curve: Evidence from Contingent Behaviour Analysis of Seafood Consumers. *Applied Economics Letters* **2008**, *15* (8), 625–628.

(5) Nguyen, Q.; Leung, P. Do Fishermen Have Different Attitudes Toward Risk? An Application of Prospect Theory to the Study of Vietnamese Fishermen. *J. Agric. Resour. Econ.* **2009**, *34* (3), 518–538.

(6) Mackay, M.; Yamazaki, S.; Jennings, S.; Sibly, H.; Putten, I.; Putten, I.; Emery, T. M. J. The Influence of Nudges on Compliance Behaviour in Recreational Fisheries: A Laboratory Experiment. **2019**. https://doi.org/10.1093/ICESJMS/FSZ020.

(7) van der Werff, E.; Steg, L.; Keizer, K. It Is a Moral Issue: The Relationship between Environmental Self-Identity, Obligation-Based Intrinsic Motivation and pro-Environmental Behaviour. *Global Environmental Change* **2013**, *23* (5), 1258–1265. https://doi.org/10.1016/j.gloenvcha.2013.07.018.

(8) *The European Landing Obligation: Reducing Discards in Complex, Multi-Species and Multi-Jurisdictional Fisheries*; Uhlmann, S. S., Ulrich, C., Kennelly, S. J., Eds.; Springer International Publishing: Cham, 2019. https://doi.org/10.1007/978-3-030-03308-8.

(9) Drupp, M. A.; Khadjavi, M.; Quaas, M. F. Truth-Telling and the Regulator. Experimental Evidence from

Commercial Fishermen. *European Economic Review* **2019**, *120*, 103310. https://doi.org/10.1016/j.euroecorev.2019.103310.

(10) Ertör-Akyazi, P. Formal versus Informal Institutions: Extraction and Earnings in Framed Field Experiments with Small-Scale Fishing Communities in Turkey. *Marine Policy* **2019**, *109*, 103673. https://doi.org/10.1016/j.marpol.2019.103673.

(11) Jodice, L. W.; Norman, W. C. Comparing Importance and Confidence for Production and Source Attributes of Seafood among Residents and Tourists in South Carolina and Florida Coastal Communities. *Appetite* **2020**, *146*, 104510. https://doi.org/10.1016/j.appet.2019.104510.

(12) Herrón, P.; Kluger, L. C.; Castellanos-Galindo, G. A.; Wolff, M.; Glaser, M. Understanding Gear Choices and Identifying Leverage Points for Sustainable Tropical Small-Scale Marine Fisheries. *Ocean & Coastal Management* **2020**, *188*, 105074. https://doi.org/10.1016/j.ocecoaman.2019.105074.

(13) Duncan, S.; Hepburn, C.; Papachristodoulou, A. Optimal Harvesting of Fish Stocks under a Time-Varying Discount Rate. *J Theor Biol* **2011**, *269* (1), 166–173. https://doi.org/10.1016/j.jtbi.2010.10.002.

(14) Ostrom, E. A General Framework for Analyzing Sustainability of Social-Ecological Systems. *Science* **2009**, *325* (5939), 419–422. https://doi.org/10.1126/science.1172133.

(15) Frey, B. S.; Benz, M.; Stutzer, A. Introducing Procedural Utility: Not Only What, but Also How Matters. *Journal of Institutional and Theoretical Economics (JITE) / Zeitschrift für die gesamte Staatswissenschaft* **2004**, *160* (3), 377–401.

(16) Bramer, W. M.; Rethlefsen, M. L.; Kleijnen, J.; Franco, O. H. Optimal Database Combinations for Literature Searches in Systematic Reviews: A Prospective Exploratory Study. *Systematic Reviews* **2017**, *6* (1), 245. https://doi.org/10.1186/s13643-017-0644-y.

Key for selection options

4 - Country

Afghanistan
Albania
Algeria
Andorra
Angola
Antigua & Deps
Argentina
Armenia
Australia
Austria
Azerbaijan
Bahamas
Bahrain
Bangladesh
Barbados
Belarus
Belgium

Belize
Benin
Bhutan
Bolivia
Bosnia Herzegovina
Botswana
Brazil
Brunei
Bulgaria
Burkina
Burundi
Cambodia
Cameroon
Canada
Cape Verde
Central African Rep
Chad
Chile
China
Colombia
Comoros
Congo
Congo {Democratic Rep}
Costa Rica
Croatia
Cuba
Cyprus
Czech Republic
Denmark
Djibouti
Dominica
Dominican Republic
East Timor
Ecuador
Egypt
El Salvador
Equatorial Guinea
Eritrea
Estonia
Ethiopia
Fiji
Finland
France
Gabon
Gambia
Georgia
Germany
Ghana
Greece

Grenada
Guatemala
Guinea
Guinea-Bissau
Guyana
Haiti
Honduras
Hungary
Iceland
India
Indonesia
Iran
Iraq
Ireland {Republic}
Israel
Italy
Ivory Coast
Jamaica
Japan
Jordan
Kazakhstan
Kenya
Kiribati
Korea North
Korea South
Kosovo
Kuwait
Kyrgyzstan
Laos
Latvia
Lebanon
Lesotho
Liberia
Libya
Liechtenstein
Lithuania
Luxembourg
Macedonia
Madagascar
Malawi
Malaysia
Maldives
Mali
Malta
Marshall Islands
Mauritania
Mauritius
Mexico
Micronesia

Moldova
Monaco
Mongolia
Montenegro
Morocco
Mozambique
Myanmar, {Burma}
Namibia
Nauru
Nepal
Netherlands
New Zealand
Nicaragua
Niger
Nigeria
Norway
Oman
Pakistan
Palau
Panama
Papua New Guinea
Paraguay
Peru
Philippines
Poland
Portugal
Qatar
Romania
Russian Federation
Rwanda
St Kitts & Nevis
St Lucia
Saint Vincent & the Grenadines
Samoa
San Marino
Sao Tome & Principe
Saudi Arabia
Senegal
Serbia
Seychelles
Sierra Leone
Singapore
Slovakia
Slovenia
Solomon Islands
Somalia
South Africa
South Sudan
Spain

Sri Lanka
Sudan
Suriname
Swaziland
Sweden
Switzerland
Syria
Taiwan
Tajikistan
Tanzania
Thailand
Togo
Tonga
Trinidad & Tobago
Tunisia
Turkey
Turkmenistan
Tuvalu
Uganda
Ukraine
United Arab Emirates
United Kingdom
United States
Uruguay
Uzbekistan
Vanuatu
Vatican City
Venezuela
Vietnam
Yemen
Zambia
Zimbabwe
