# Supplementary material for: Behavioural economics in fisheries: A systematic review protocol
Source: PLoS One. 2021 Aug 26;16(8):e0255333. doi: 10.1371/journal.pone.0255333 (PMC8389455; doi:10.1371/journal.pone.0255333)
Supplement: S3 File — (PDF) [file pone.0255333.s008.pdf]

S3 File: Details of Boolean search string for each databases

Web of Science – Core Collection
Within the Web of Science Core Collection four individual searches will be carried out for each thematic group of the keywords using the "TS = topic" field tag. The four searches will then be combined using the "AND" combine function which is available through the database.

Search strings:
Search #1: TS = *(behavio* AND fisher*)*
Search #2: TS = *(human OR soci*)*
Search #3: TS = *(interve* OR experiment* OR mechanis* OR observ* OR chang*)*
Search #4: TS = (*anchoring OR arbitrage OR (being watched) OR (behavio* econom*) OR bias OR (bounded rationality) OR (co*creation) OR (cognitive bias*) OR (collaborative manag*) OR (co*management) OR complian OR (co*operat*) OR (crowding out) OR (cultural norm*) OR decoy OR default OR (disposition effect) OR (cognitive dissonance) OR labelling OR educat* OR empower OR fairness OR framing OR (game theory) OR heuristic OR (hyperbolic discounting) OR identity OR incentiv* OR (intrinsic motivation*) OR leadership OR (loss aversion) OR management OR (mental accounting) OR mechanism* OR (msc cert*) OR nudg* OR (participatory manag*) OR preference OR (present bias) OR priming OR (probability weighting) OR (procedural utility) OR (prospect theory) OR (satisfaction delay) OR (self*perception) OR (self*serving) OR (self*theory) OR (soci* categor*) OR (soci* learning) OR (social norm*) OR (social proof) OR (soci* factor*) OR (soci* driver*) OR status-quo OR (sunk cost) OR trust OR values)*

ProQuest – Social Science Collection
The selected databases of the Social Science Collection of ProQuest will be searched using the "noft = anywhere except full text" field tag and one continuous Boolean search string.

Search string:
Noft((behavio AND fisher*) AND (human OR soci*) AND (interve* OR experiment* OR mechanis* OR observ* OR chang*) AND (anchoring OR arbitrage OR (being watched) OR (behavio* econom*) OR bias OR (bounded rationality) OR (co*creation) OR (cognitive bias*) OR (collaborative manag*) OR (co*management) OR complian OR (co*operat*) OR (crowding out) OR (cultural norm*) OR decoy OR default OR (disposition effect) OR (cognitive dissonance) OR labelling OR educat* OR empower OR fairness OR framing OR (game theory) OR heuristic OR (hyperbolic discounting) OR identity OR incentiv* OR (intrinsic motivation*) OR leadership OR (loss aversion) OR management OR (mental accounting) OR mechanism* OR (msc cert*) OR nudg* OR (participatory manag*) OR preference OR (present bias) OR priming OR (probability weighting) OR (procedural utility) OR (prospect theory) OR (satisfaction delay) OR (self*perception) OR (self*serving) OR (self*theory) OR (soci* categor*) OR (soci* learning) OR (social norm*) OR (social proof) OR (soci* factor*) OR (soci* driver*) OR status-quo OR (sunk cost) OR trust OR values))*

EconLit
Within EBSCO, the EconLit database will be searched using one continuous Boolean search string. No field tags similar to "topic" or "anywhere except full text" are available within EconLit and therefore abstracts, titles and text will be screened.

Search string:
(behavio AND fisher*) AND (human OR soci*) AND (interve* OR experiment* OR mechanis* OR observ* OR chang*) AND (anchoring OR arbitrage OR (being watched) OR (behavio* econom*) OR bias OR (bounded rationality) OR (co*creation) OR (cognitive bias*) OR (collaborative manag*) OR (co*management) OR complian OR (co*operat*) OR (crowding out) OR (cultural norm*) OR decoy OR default OR (disposition effect) OR (cognitive dissonance) OR labelling OR educat* OR empower OR fairness OR framing OR (game theory) OR heuristic OR (hyperbolic discounting) OR*

identity OR incentiv OR (intrinsic motivation*) OR leadership OR (loss aversion) OR management OR (mental accounting) OR mechanism* OR (msc cert*) OR nudg* OR (participatory manag*) OR preference OR (present bias) OR priming OR (probability weighting) OR (procedural utility) OR (prospect theory) OR (satisfaction delay) OR (self*perception) OR (self*serving) OR (self*theory) OR (soci* categor*) OR (soci* learning) OR (social norm*) OR (social proof) OR (soci* factor*) OR (soci* driver*) OR status-quo OR (sunk cost) OR trust OR values*
