# Supplementary material for: Behavioural economics in fisheries: A systematic review protocol
Source: PLoS One. 2021 Aug 26;16(8):e0255333. doi: 10.1371/journal.pone.0255333 (PMC8389455; doi:10.1371/journal.pone.0255333)
Supplement: S4 File — (PDF) [file pone.0255333.s009.pdf]

Full Text Screening Form

Reviewer and Article Information

Welcome to the behavioural economics systematic review text screening form.

Remember that this form expires after 1-2 hours. We therefore would recommend that you first read the entire paper and then populate this form.

IMPORTANTLY, please make sure that if you are copying text directly from the paper into this form to indicate this by putting the inserted text into quotation marks (") and stating the relevant page numper within the paper (p.x).

Name
Please select your name from the list. ✱ *Required*

- ◯ Alina
- ◯ Amanda
- ◯ Julie
- ◯ Mary
- ◯ Ingrid
- ◯ Sarah
- ◯ Debbi
- ◯ Leyre
- ◯ Other

If you selected Other, please specify:

____

DOI

Please enter the article doi (starting with "10..."). ✱ *Required*

Title
Please enter the article title. ✱ *Required*

Full Text Inclusion/Exclusion
After reading the full article, do you think it should be included or excluded from the screening?

If in doubt consult the below key-eligibility criteria list.
- Population*: Is it fishers?*
- Population*: Is it marine (i.e. not fresh water)?*
- Intervention: *Is the main driver investigated NOT rules/compliance or market? (i.e. something else, compliance and market can be included but not that main)?*
- Outcomes: *Does it a) address reasons behind actual behaviour in reality or b) propose a way behaviour could be changed theoretically?*

Please make sure that when you select "exclude" or "unsure" you still need to click "next" at the bottom of the page, otherwise your response will not be recorded. ✱ *Required*

- Include
- Exclude
- Unsure

Reason for Exclusion

Please give the reason for exclusion. ✱ *Required*

Reason for Selecting 'Unsure'

Please give the reason for why you are unsure about whether or not the article should be included.
✱ *Required*

Article Synthesis Form

Country
Select the country (or mutltiple counties where applicable) in which the study took place. If the country is not known or it is unclear where the study took place select "unknown". ✱ *Required*

- ☐ Unknown
- ☐ No specific geographical focus
- ☐ Afghanistan
- ☐ Åland Islands
- ☐ Albania
- ☐ Algeria
- ☐ American Samoa
- ☐ Andorra
- ☐ Angola
- ☐ Anguilla
- ☐ Antarctica
- ☐ Antigua and Barbuda
- ☐ Argentina
- ☐ Armenia
- ☐ Aruba
- ☐ Australia
- ☐ Austria
- ☐ Azerbaijan
- ☐ Bahrain
- ☐ Bahamas
- ☐ Bangladesh
- ☐ Barbados
- ☐ Belarus
- ☐ Belgium
- ☐ Belize
- ☐ Benin
- ☐ Bermuda
- ☐ Bhutan
- ☐ Bolivia, Plurinational State of
- ☐ Bonaire, Sint Eustatius and Saba
- ☐ Bosnia and Herzegovina
- ☐ Botswana
- ☐ Bouvet Island
- ☐ Brazil
- ☐ British Indian Ocean Territory
- ☐ Brunei Darussalam
- ☐ Bulgaria
- ☐ Burkina Faso
- ☐ Burundi
- ☐ Cambodia
- ☐ Cameroon
- ☐ Canada
- ☐ Cape Verde
- ☐ Cayman Islands
- ☐ Central African Republic
- ☐ Chad
- ☐ Chile
- ☐ China
- ☐ Christmas Island
- ☐ Cocos (Keeling) Islands
- ☐ Colombia
- ☐ Comoros
- ☐ Congo
- ☐ Congo, the Democratic Republic of the
- ☐ Cook Islands
- ☐ Costa Rica
- ☐ Côte d'Ivoire
- ☐ Croatia
- ☐ Cuba
- ☐ Curaçao
- ☐ Cyprus
- ☐ Czech Republic
- ☐ Denmark
- ☐ Djibouti
- ☐ Dominica
- ☐ Dominican Republic
- ☐ Ecuador
- ☐ Egypt
- ☐ El Salvador
- ☐ Equatorial Guinea
- ☐ Eritrea
- ☐ Estonia
- ☐ Ethiopia
- ☐ Falkland Islands (Malvinas)
- ☐ Faroe Islands
- ☐ Fiji
- ☐ Finland
- ☐ France

☐ French Guiana	☐ French Polynesia	☐ French Southern Territories
☐ Gabon	☐ Gambia	☐ Georgia
☐ Germany	☐ Ghana	☐ Gibraltar
☐ Greece	☐ Greenland	☐ Grenada
☐ Guadeloupe	☐ Guam	☐ Guatemala
☐ Guernsey	☐ Guinea	☐ Guinea-Bissau
☐ Guyana	☐ Haiti	☐ Heard Island and McDonald Islands
☐ Holy See (Vatican City State)	☐ Honduras	☐ Hong Kong
☐ Hungary	☐ Iceland	☐ India
☐ Indonesia	☐ Iran, Islamic Republic of	☐ Iraq
☐ Ireland	☐ Isle of Man	☐ Israel
☐ Italy	☐ Jamaica	☐ Japan
☐ Jersey	☐ Jordan	☐ Kazakhstan
☐ Kenya	☐ Kiribati	☐ Korea, Democratic People's Republic of
☐ Korea, Republic of	☐ Kuwait	☐ Kyrgyzstan
☐ Lao People's Democratic Republic	☐ Latvia	☐ Lebanon
☐ Lesotho	☐ Liberia	☐ Libya
☐ Liechtenstein	☐ Lithuania	☐ Luxembourg
☐ Macao	☐ Macedonia, the Former Yugoslav Republic of	☐ Madagascar
☐ Malawi	☐ Malaysia	☐ Maldives
☐ Mali	☐ Malta	☐ Marshall Islands
☐ Martinique	☐ Mauritania	☐ Mauritius
☐ Mayotte	☐ Mexico	☐ Micronesia, Federated States of
☐ Moldova, Republic of	☐ Monaco	☐ Mongolia
☐ Montenegro	☐ Montserrat	☐ Morocco
☐ Mozambique	☐ Myanmar	☐ Namibia
☐ Nauru	☐ Nepal	☐ Netherlands
☐ New Caledonia	☐ New Zealand	☐ Nicaragua
☐ Niger	☐ Nigeria	☐ Niue
☐ Norfolk Island	☐ Northern Mariana Islands	☐ Norway

- Oman
- Pakistan
- Palau
- Palestine, State of
- Panama
- Papua New Guinea
- Paraguay
- Peru
- Philippines
- Pitcairn
- Poland
- Portugal
- Puerto Rico
- Qatar
- Réunion
- Romania
- Russian Federation
- Rwanda
- Saint Barthélemy
- Saint Helena, Ascension and Tristan da Cunha
- Saint Kitts and Nevis
- Saint Lucia
- Saint Martin (French part)
- Saint Pierre and Miquelon
- Saint Vincent and the Grenadines
- Samoa
- San Marino
- Sao Tome and Principe
- Saudi Arabia
- Senegal
- Serbia
- Seychelles
- Sierra Leone
- Singapore
- Sint Maarten (Dutch part)
- Slovakia
- Slovenia
- Solomon Islands
- Somalia
- South Africa
- South Georgia and the South Sandwich Islands
- South Sudan
- Spain
- Sri Lanka
- Sudan
- Suriname
- Svalbard and Jan Mayen
- Swaziland
- Sweden
- Switzerland
- Syrian Arab Republic
- Taiwan, Province of China
- Tajikistan
- Tanzania, United Republic of
- Thailand
- Timor-Leste
- Togo
- Tokelau
- Tonga
- Trinidad and Tobago
- Tunisia
- Turkey
- Turkmenistan
- Turks and Caicos Islands
- Tuvalu
- Uganda
- Ukraine
- United Arab Emirates
- United Kingdom
- United States
- United States Minor Outlying Islands
- Uruguay
- Uzbekistan
- Vanuatu
- Venezuela, Bolivarian Republic of
- Viet Nam
- Virgin Islands, British
- Virgin Islands, U.S.
- Wallis and Futuna
- Western Sahara
- Yemen
- Zambia
- Zimbabwe

Continent

Select continent (or mutltiple continents where applicable) in which the study took place. If the continent is not known or it is unclear where the study took place select "unknown". ✱ *Required*

- ☐ Unknown
- ☐ No specific geographical focus
- ☐ Asia
- ☐ Africa
- ☐ North America
- ☐ South America
- ☐ Antarctica
- ☐ Europe
- ☐ Australia

Seas and Oceans

Using the map of global seas and oceans please select in which ocean(s)/sea(s) the study took place. If the ocean/sea is not known or it is unclear where the study took place select "unknown".

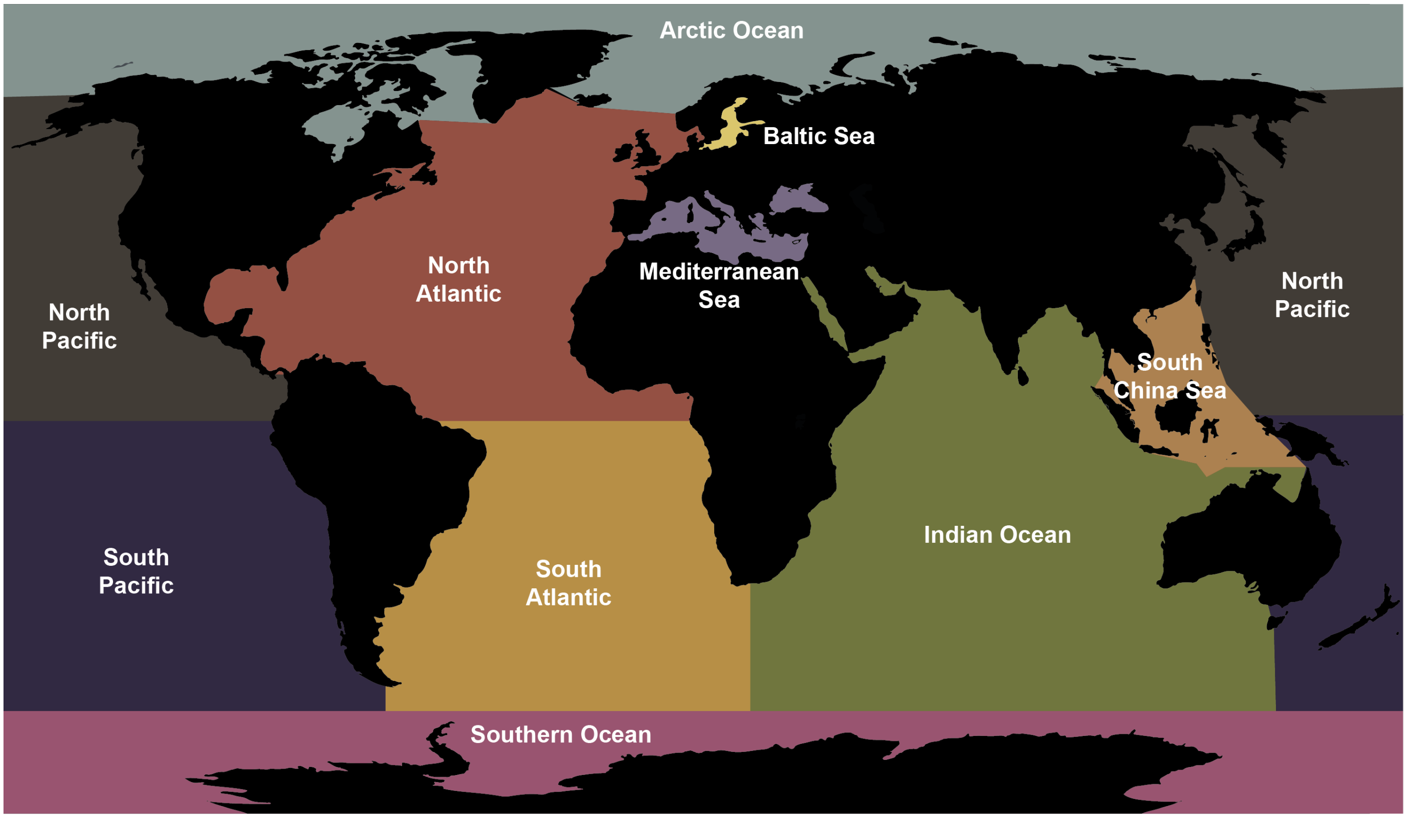

✱ *Required*

- ☐ Unknown
- ☐ No specific geographical focus
- ☐ Arctic Ocean
- ☐ Baltic Sea
- ☐ Indian Ocean
- ☐ Mediterranean Sea
- ☐ North Atlantic
- ☐ North Pacific
- ☐ South Atlantic
- ☐ South China Sea
- ☐ South Pacific
- ☐ Southern Ocean

Geographical Scale
Select scale at which the study took place. For theoretical studies which did not investigate specific fisheries scale select n/a.

Local is defined as "*relating or restricted to a particular area or one's neighbourhood*". Regional is defined as "*an area, especially part of a country or the world having definable characteristics but not always fixed boundaries*". ✱ *Required*

- ☐ n/a
- ☐ Unclear
- ☐ Local
- ☐ Regional
- ☐ National
- ☐ Global

Fisheries Scale
Please select the fisheries scale(s) which was(were) the focus of the study as defined in the answer options. For theoretical studies which did not target a specific fisheries select n/a. ✱ *Required*

- ☐ n/a
- ☐ Unclear
- ☐ Recreational
- ☐ Artisanal (<10m or low output or old/traditional technique)
- ☐ Small (<20m)
- ☐ Large (>20m)

Gear Type
Please select gear type(s) used by fishers in the study (for info: https://www.msc.org/what-we-are-doing/our-approach/fishing-methods-and-gear-types). If the gear type is not listed use "other" box, if unclear select "unknown". For theoretical studies which did not investigate specific fisheries scale select n/a. ✱ *Required*

- ☐ n/a
- ☐ Unknown
- ☐ Demersal or bottom trawl
- ☐ Gillnets
- ☐ Longlines
- ☐ Purse seine
- ☐ Pole and line
- ☐ Pots and traps
- ☐ Dredges
- ☐ Pelagic or midwater trawl
- ☐ Bottom line
- ☐ Speargun and traps
- ☐ Gillnets seabed to surface
- ☐ Seine nets on seabed
- ☐ Bait net
- ☐ Other

If you selected Other, please specify:

Fisheries Commercial Group
Select what fisheries commercial group the study is targeting. For theoretical studies which did not investigate specific fisheries scale select n/a. ✱ *Required*

- ☐ n/a
- ☐ Unclear
- ☐ Perch-likes
- ☐ Flatfishes
- ☐ Cod-likes
- ☐ Herring-likes
- ☐ Anchovies
- ☐ Crustaceans
- ☐ Tuna & billfishes
- ☐ Molluscs
- ☐ Salmon, smelts etc.
- ☐ Scorpionfishes
- ☐ Sharks & Rays

- [ ] Other fishes and invertebrates

Depleted Fish Stocks
Below is a list of fish stocks identified by FAO as falling into its "worst" category, "depleted" (http://www.fao.org/newsroom/common/ecg/1000505/en/stocks.pdf). If the study concerns any of the below listed fish stocks please select the relevant one.

Atlantic Species *Optional*

- [ ] Cods
- [ ] Haddocks
- [ ] Salmons, trouts and smelts
- [ ] Hakes
- [ ] Whiting
- [ ] Atlantic Bluefin Tuna
- [ ] Geelbek Croaker
- [ ] Red steenbras

Pacific Species *Optional*

- [ ] Southern Bluefin Tuna
- [ ] North Pacific Hake
- [ ] Shrimps and Prawns
- [ ] Eastern Pacific Bonito
- [ ] South Pacific Hake

Indian Ocean *Optional*

- [ ] Southern Bluefin Tuna

Mediterranean *Optional*

- ☐ Albacore
- ☐ Bluefin Tuna
- ☐ Bonito
- ☐ Azov Sea Sprat
- ☐ European Sprat
- ☐ Sardinellas
- ☐ Shads
- ☐ Whiting

Southern Ocean *Optional*

- ☐ Antarctic Rockcods
- ☐ Blackfin Icefish
- ☐ Patagonian Toothfish
- ☐ Mackerel Icefish

Factor Type
In the below is a list of the different types of factors which have been identified by Andrews et al. 2020 to affect fisher behaviour.

Please select which type of factors were found to affect fisher behaviour in the reviewed study. ✱
Required

- ☐ Demographic (e.g. age, education, income)
- ☐ Psychosocial (e.g. values, attitudes, risk perception)
- ☐ Environmental (e.g. catchability, weather, habitat condition)
- ☐ Economic (e.g. vessel or gear, poverty, expected value of landings)
- ☐ Socio-cultural (e.g. tradition, conflict within communities, social norms)
- ☐ Governance (e.g. regulatory strength, access to fishing groups, incentives)

Behavioural Economics Mechanisms and Interventions

From the lists below select which behavioural economics mechanism(s) and intervention(s) have been investigated in the study. As a guide refer to this table which has resulted from a pre-scoping exercise with the WGMARS stakeholders

Please note, that this table is unlikely to be complete and add any other mechanisms and interventions you may have identified in the "other" box.

Please select mechanism(s). ✱ *Required*

- ☐ Unclear
- ☐ Reference dependence/Shifting baselines
- ☐ Anchoring effect
- ☐ Non-linear probability weighting
- ☐ Self-image
- ☐ Priming
- ☐ Framing
- ☐ Hedonic Framing
- ☐ Paradox of Choice
- ☐ Ego depletion
- ☐ Effect of being watched/panopticism
- ☐ Status-quo bias
- ☐ Decoy effect
- ☐ Availability heuristic
- ☐ Belief bias
- ☐ Confirmation bias
- ☐ Gamblers fallacy
- ☐ Illusion of validity
- ☐ Ostrich effect
- ☐ Post-purchase rationalization
- ☐ Risk compensation
- ☐ Clustering illusion
- ☐ Endowment Effect / sunk cost bias / disposition effect
- ☐ Present bias / Hyperbolic discounting
- ☐ Injunctive/normative social norms / social license to operate
- ☐ Descriptive norms
- ☐ Bandwagon/snob effect
- ☐ Blind spot bias
- ☐ Courtesy bias
- ☐ Reactive devaluation
- ☐ Stereotyping
- ☐ Other

If you selected Other, please specify:

Please select intervention(s): *Optional*

- ☐ n/a
- ☐ Unclear
- ☐ Incentive
- ☐ Punishment
- ☐ Nudge
- ☐ Co-production
- ☐ Education
- ☐ Other

If you selected Other, please specify:

Instigating Party
Please select the instigating party of the study. For this please select any party which was involved in the study.

E.g.: If the study was financed by an NGO, carried out by scientists based at an institution and executed by policy makers, please select all of these in the list below. ✱ *Required*

- ☐ n/a
- ☐ Unknown
- ☐ Scientists
- ☐ Policy makers
- ☐ Fishers themselves
- ☐ Marketing company
- ☐ NGO or other organisation
- ☐ Community
- ☐ Other

If you selected Other, please specify:

Why intervene? What is the Problem?
Please state the specific problem this paper addresses and/or the reason why this study was conducted as stated in the introduction. Please be as specific as possible; for example, instead of just writing 'overfishing' narrow it down to something like 'compliance with a newly introduced rule on ...' or 'investigate declining participation by fishers in ... programme'. ✱ *Required*

Method
Please select the type of method applied in the study.

For guidance refer to the explanation and examples below.
Experiment - lab: *Where fishers are taken out of their common environment and are asked to perform a (theoretical) task. E.g.: Mackay et al. 2020: The influence of nudges on compliance behaviour in recreational fisheries: a laboratory experiment*
Experiment - field: *Where an intervention is introduced and it is observed what happens. E.g.: McDonald et al. 2020: Catalyzing sustainable fisheries management through behaviour change interventions.*
Descriptive - qualitative: *where fishers are interviewed in rich detail to investigate how a potential mechanims may be working or how a rule may be effecting someone with the aim to qualify relationships between different things.*
Descriptive - quantitative: *Where surveys are done on numbers (e.g. logbooks) or surveys are carried out on a numerical scale.*
Descriptive - mixed methods: *Where one may ask fishers where they fish and why they fish then but then also use numerical data (e.g. logbooks). E.g.: Schadeberg et al. 2021*
Model*: Where a representation of the reality is produced to predict or understand what happens in the futue/on a bigger scale/in another place.* ✱ *Required*

- ☐ Experiment - lab
- ☐ Experiment - field
- ☐ Descriptive - qualitative
- ☐ Descriptive - quantitative

- [ ] Descriptive - mixed methods
- [ ] Model

Behavioural Level - Intervention
Please indicate the behavioural level at which the intervention took place (where applicable). ✱ *Required*

- ◯ n/a
- ◯ Unclear
- ◯ Individuals and Groups
- ◯ Individuals
- ◯ Groups

Behavioural Level - Observation
Please indicate the behavioural level at which the measurement took place. ✱ *Required*

- ◯ Unclear
- ◯ Individuals and Groups
- ◯ Individuals
- ◯ Groups

Notes/Comments on Method *Optional*

Intervention
Please describe the discussed interventions/change in behaviour (e.g. sending text message to fisher) where applicable. *Optional*

Effect
Please ***briefly*** (few words) describe the effect of the intervention (e.g. choice of area, change in values, compliance) where applicable.

For instance: “the intervention (a flyer campaign triggering injunctive social norms) reduced mammal bycatch in the study population (fishers in Atlantis)" *Optional*

Does the study measure effects in the short term only (i.e. immediately after the intervention) or do the researchers measure long-term effects, or both? *Optional*

- ○ Short-term only
- ○ Long-term (more than 1 year after initial intervention)
- ○ Short term with a long-term follow-up
- ○ Unclear or not mentioned

If necessary, please provide further clarification on the timescale of the effect measure. *Optional*

Effect Size of Intervention
Please describe the effect size of the intervention where applicable.

For instance: "mammal bycatch was reduced by 20% in two years" *Optional*

Unintended Effects

Were any unintended effects observed in the study? ✱ *Required*

- Unclear
- Yes
- No

Please specify any unintended effects.

Relevance to Fisheries ✱ *Required*

- Unclear
- Study in real-world context (fishers in situ)
- Not in situ but related to, and involving fishers (e.g., Drupp et al. 2019; doi:10.1016/j.euroecorev.2019.103310)

Relationship between Fishers and Managers

What is your impression of what the relationship between the fishers and managers was before the study was conducted? Please try to gauge this to your best knowledge and only use unclear when realy necessary. ✱ *Required*

- n/a
- Unclear

- Good/Trust
- Indifferent
- Bad/Distrust

Relationship between Fishers and Managers over Time

Has the relationship between fishers and managers changed over the course of the study or intervention and beyond? ✱ *Required*

- Unclear
- Unchanged
- Improved
- Worsened

Social Outcome

Were social outcomes considered in the study? ✱ *Required*

- Yes
- No

Please described the social outcome(s) and any emperical evidence provided.

Economic Outcome

Were economic outcomes considered in the study? ✱ *Required*

- Yes
- No

Please described the economic outcome(s) and any emperical evidence provided.

Environmental Outcome
Were environmental outcomes considered in the study? ✱ *Required*

- Yes
- No

Please described the environmental outcome(s) and any emperical evidence provided.

Management and Policy
Does the article highlight any management strategies or policy recommendations which have real-world application in the future? ✱ *Required*

- Yes
- No

Please outline the management strategies and/or policy recommendations in the below.

Ethics

Were ethical implications of research and policy actions which influencing fisher behaviour considered?
✱ *Required*

- ○ Unclear
- ○ Yes
- ○ No

Please provide some detail on how ethical implications were considered in the study.

Please write a one-sentence conclusion of the study. ✱ *Required*

Is there something about this article that makes it unique/important/interesting? *Optional*

Does the article mention any relevant research projects, studies or theoretical concepts which would be worth considering in the discussion of our review?

Please provide a weblink and/or doi references.

E.g.: Fish Forever Project (https://rare.org/program/fish-forever/) OR article on social punishment which may lead to cooperation (doi: 10.1016/0162-3095(92)90032-Y) *Optional*
